# Niche-induced extramedullary hematopoiesis in the spleen is regulated by the transcription factor Tlx1

**DOI:** 10.1038/s41598-018-26693-x

**Published:** 2018-05-29

**Authors:** Akihisa Oda, Toshiki Tezuka, Yuta Ueno, Shoko Hosoda, Yusuke Amemiya, Chihiro Notsu, Toru Kasahara, Chiharu Nishiyama, Ryo Goitsuka

**Affiliations:** 1 Division of Development and Aging, Research Institute for Biomedical Sciences, Tokyo University of Science, Chiba, Japan; 2 Laboratory of Molecular Biology and Immunology, Department of Biological Science and Technology, Tokyo University of Science, Tokyo, Japan; 3 Center for Animal Disease Models, Tokyo University of Science, Chiba, Japan; 4 Imaging Frontier Center, Tokyo University of Science, Chiba, Japan

## Abstract

Extramedullary hematopoiesis (EMH) in postnatal life is a pathological process in which the differentiation of hematopoietic stem/progenitor cells (HSPCs) occurs outside the bone marrow (BM) to respond to hematopoietic emergencies. The spleen is a major site for EMH; however, the cellular and molecular nature of the stromal cell components supporting HSPC maintenance, the niche for EMH in the spleen remain poorly understood compared to the growing understanding of the BM niche at the steady-state as well as in emergency hematopoiesis. In the present study, we demonstrate that mesenchymal progenitor-like cells expressing Tlx1, an essential transcription factor for spleen organogenesis, and selectively localized in the perifollicular region of the red pulp of the spleen, are a major source of HSPC niche factors. Consistently, overexpression of Tlx1 *in situ* induces EMH, which is associated with mobilization of HSPC into the circulation and their recruitment into the spleen where they proliferate and differentiate. The alterations in the splenic microenvironment induced by Tlx1 overexpression *in situ* phenocopy lipopolysaccharide (LPS)-induced EMH, and the conditional loss of Tlx1 abolished LPS-induced splenic EMH. These findings indicate that activation of Tlx1 expression in the postnatal splenic mesenchymal cells is critical for the development of splenic EMH.

## Introduction

Hematopoiesis is a highly orchestrated process that generates multi-lineage blood cells from a small pool of hematopoietic stem/progenitor cells (HSPCs) through a successive series of increasingly lineage-restricted intermediate progenitors^1^. Under steady state conditions throughout postnatal life, HSPCs are mainly localized within the bone marrow (BM) in specialized microenvironments termed niches, where signals from other cells in the niche maintain their survival and functions^2,3^. However, under emergency conditions, such as inflammation, anemia, myelofibrosis and other pathologic situations where there is bone marrow failure, hematopoiesis occurs outside the BM, including the spleen and liver, as a result of pathophysiological alterations in HSPCs as well as the ectopic emergence of their niche in these tissues, a process called extramedullary hematopoiesis (EMH)^4,5^.

Given that splenomegaly is the most frequently observed feature of EMH, the spleen functions not only as a secondary lymphoid organ but also as a hematopoietic organ^6^. The spleen is comprised of spatially and functionally distinct compartments; the white pulp, surrounded by the marginal zone, contains mainly lymphoid cells for immune responses and the red pulp, consisting of venous sinusoids and mesenchymal cells. At homeostasis the red pulp functions in erythrocyte turnover^7^ and as reservoir of macrophages and erythrocytes for a rapid supply into the circulation in an emergency^8–10^. The red pulp also serves as a site for EMH with a concomitant expansion of the stromal cell compartment^11^. In this regard, as in the fetal liver, hematopoiesis occurs in the fetal spleen around embryonic day E14.5 in mice, at which time point erythropoiesis and myelopoiesis predominate in the presumptive red pulp, persisting until one week after birth^12,13^, while the structure of the white pulp surrounded by the marginal sinus gradually becomes organized with the proper positioning of T and B cell areas after birth^14^. In addition, it has been reported that the number of colony-forming hematopoietic progenitors in the spleen increases, peaking at two weeks of age in mice^15^, and that HSPCs are recruited to the spleen during the neonatal period^16^. Furthermore, HSPCs have been identified in close association with the endothelium of red pulp sinuses in postnatal mice^17^. Thus, the red pulp area of the spleen in mice, unlike in humans, by retaining residual hematopoietic activity during the postnatal period is a favorable site for a HSPC niche for EMH^4,5^. However, the cellular and molecular nature of the components organizing the HSPC niche for EMH in the spleen remain poorly understood, compared to the growing understanding of the BM niche at the steady-state as well as in emergency hematopoiesis^2,18^.

Several transcription factors expressed in embryonic spleen mesenchymal cells, such as Pbx1, WT1, Tcf21 and Nk3.2., have been shown to be required for spleen organogenesis, as their deficiency causes spleen agenesis or hypoplasia, in association with other organ defects^19–22^. Among these transcription factors, Tlx1 is expressed in mesenchymal cells that are relatively restricted to the spleen primordium, and probably as a result, the asplenia occurs without detectable abnormalities in other organs of *Tlx1* knockout mice^23,24^. Taking an advantage of the selective Tlx1 expression in spleen mesenchymal cells, we have recently generated mice harboring a mutant *Tlx1* gene allele, in which *CreER* and *Venus* genes are knocked into the first exon of the *Tlx1* gene (*Tlx1*^*CreER-Venus*^), for conditional *in vivo* genetic manipulation and lineage tracing of spleen mesenchymal cells. We demonstrated that Tlx1 is required for cell fate determination of mesenchymal cells of the spleen anlage, as Tlx1-deficient progeny in the embryonic spleen anlage, cells in which Tlx1 was once transcriptionally activated, become dorsal pancreatic mesenchymal cells^25^.

In the present study, we examined the phenotype and function of Tlx1-expressing mesenchymal cells in the postnatal spleen and also the function of Tlx1 itself in these cells by using *Tlx1*^*CreER-Venus*^ mice and demonstrated that Tlx1-expressing cells are a component of the HSPC niche in the spleen. Moreover, high levels of Tlx1 expression *in situ* are sufficient to induce EMH and are also required for the recruitment of HSPCs to the spleen in lipopolysaccharide (LPS)-induced EMH.

## Results

### Tlx1 selectively marks mesenchymal progenitor-like cells enriched in HSPC niche factors in the postnatal spleen

**Figure 1:**
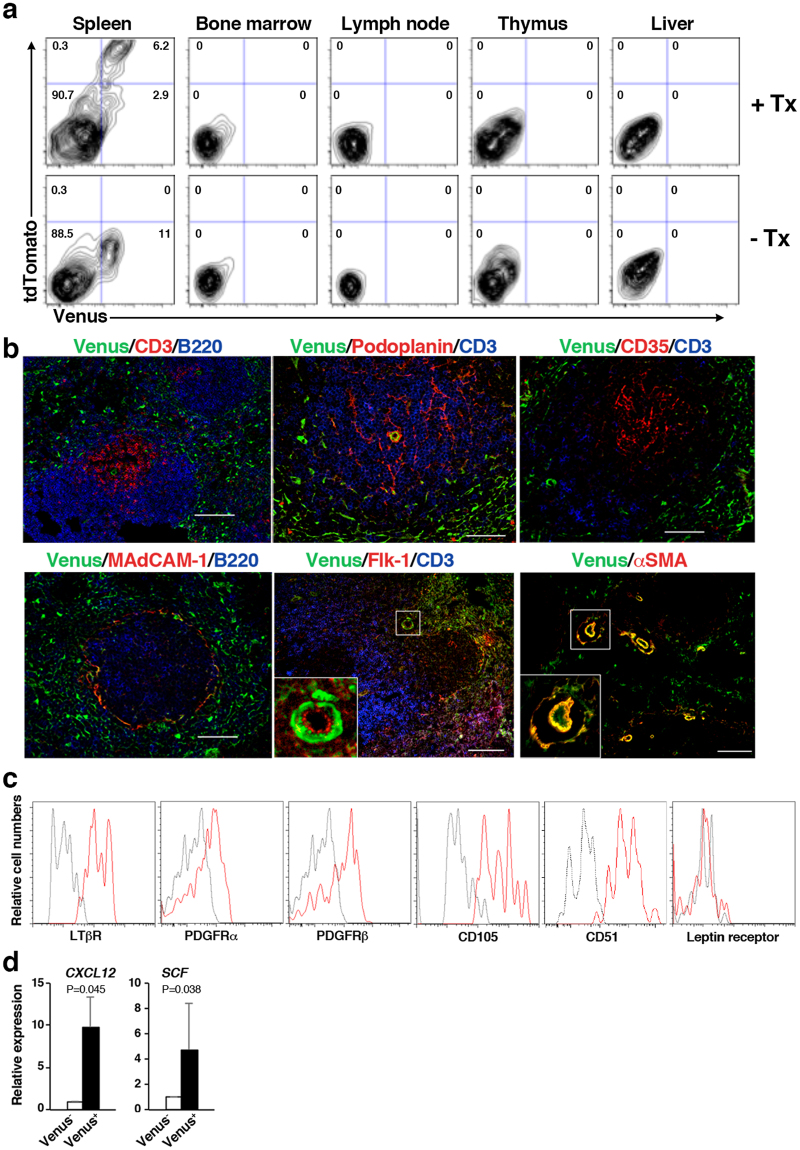
Tlx1 selectively marks mesenchymal progenitor-like cells enriched in HSPC niche factors in the postnatal spleen. (**a**) Representative flow cytometric profiles of CD45^−^ Ter119^−^ CD31^−^ stromal cells in the spleen, BM, lymph node, thymus and liver from *Tlx1*^*CreER-Venus*^*; R26*^*tdTomato*^ mice (4-week-old) with (upper panels) or without (lower panels) tamoxifen (Tx) treatment (24 hours after the final treatment). Gates used to identify Venus^+^ and tdTomato^+^ cell populations are outlined, and numbers above outlined areas indicate percent events in each gate (mean ± SD, n = 3). Detailed gating strategy is provided in Fig. S1. (**b**) Representative tissue section images of Venus^+^ cells in the spleen of 4-week-old *Tlx1*^*CreER-Venus*^ mice. Tissue sections were stained with the indicated antibody combinations. Inserted rectangles represent a higher magnification image. Scale bars indicate 100 μm. (n = 3). (**c)** Representative flow cytometric histograms showing surface marker expression on tdTomato^+^ cells of the spleen from *Tlx1*^*CreER-Venus*^*; R26*^*tdTomato*^ mice treated with tamoxifen, as in (**a**). (n = 3). (**d)** Expression of *CXCL12* and *SCF* mRNA in Venus^+^ cells among CD45^−^Ter119^−^ stromal cells from the spleen of *Tlx1*^*CreER*^ mice. (mean ± SD, n = 3). Data were normalized to β*-actin* and the level of mRNA transcripts in Venus^−^ cells was arbitrarily set to 1.

We have previously demonstrated that expression of Venus and CreER strictly recapitulates Tlx1-expressing cells among CD45^−^ Ter119^−^ non-hematopoietic stromal cells in the neonatal spleen of *Tlx1*^*CreER-Venus*^*; Rosa26 (R26)*^*tdTomato*^ mice^25^. To further confirm that the same is true in the postnatal spleen and to address whether Tlx1 is expressed in other hematopoietic organs, we administered tamoxifen into *Tlx1*^*CreER-Venus*^*; R26*^*tdTomato*^ mice (4-week-old) by intragastric gavage on 3 consecutive days and then examined Venus and tdTomato expression 24 hours after the final treatment. As shown in Fig. 1a, a small population of CD45^−^Ter119^−^CD31^−^ cells in the spleen was both Venus- and tdTomato-positive, whereas no Venus or tdTomato fluorescence was observed in the BM, lymph node, thymus or liver. Furthermore, Venus and tdTomato expression were both undetectable in CD45^+^Ter119^+^ CD31^+^ hematopoietic and vascular endothelial cells (Fig. S1). These findings indicate that *Tlx1*^*CreER-Venus*^ marks a stromal cell component that is unique to the postnatal spleen.

To characterize the Tlx1-expressing stromal cells of the spleen in more detail, we carried out immunohistochemical analyses by using antibodies to previously known spleen stromal cell markers combined with anti-GFP antibody for detecting Venus expression. The majority of Venus-positive cells were scattered outside the white pulp, which contains CD3^+^ T cells and B220^+^ B cells, and did not express podoplanin, CD35 or MAdCAM-1, which are known to be specific markers of fibroblastic reticular cells (FRCs), follicular dendritic cells (FDCs) and marginal reticular cells (MRCs), respectively (Fig. 1b). In addition, αSMA-positive perivascular mural cells surrounding CD31^+^ central arterioles expressed Venus. We also examined expression of mesenchymal cell markers on Venus^+^ tdTomato^+^ cells and found that they were positive for lymphotoxin receptor β (LTβR), platelet-derived growth factor receptor (PDGFR) α and β, CD105 and CD51, but were negative for leptin receptor (Fig. 1c). Given the similarity of the surface marker expression on Venus^+^ tdTomato^+^ spleen cells to the mesenchymal cells comprising the BM hematopoietic niche, with an exception of the leptin receptor, we examined hematopoietic niche factor expression by Venus^+^ cells. As expected, among CD45^−^Ter119^−^ non-hematopoietic cells in the spleen, Venus^+^ cells expressed significantly higher levels of *CXCL12* and *SCF* mRNA than Venus^−^ cells (Fig. 1d). Taken together, these findings strongly suggest that Tlx1-expressing cells are the mesenchymal progenitor-like cells with the potential to serve as the hematopoietic niche selectively in the spleen.

### High levels of Tlx1 expression in the spleen *in situ* induce EMH

**Figure 2:**
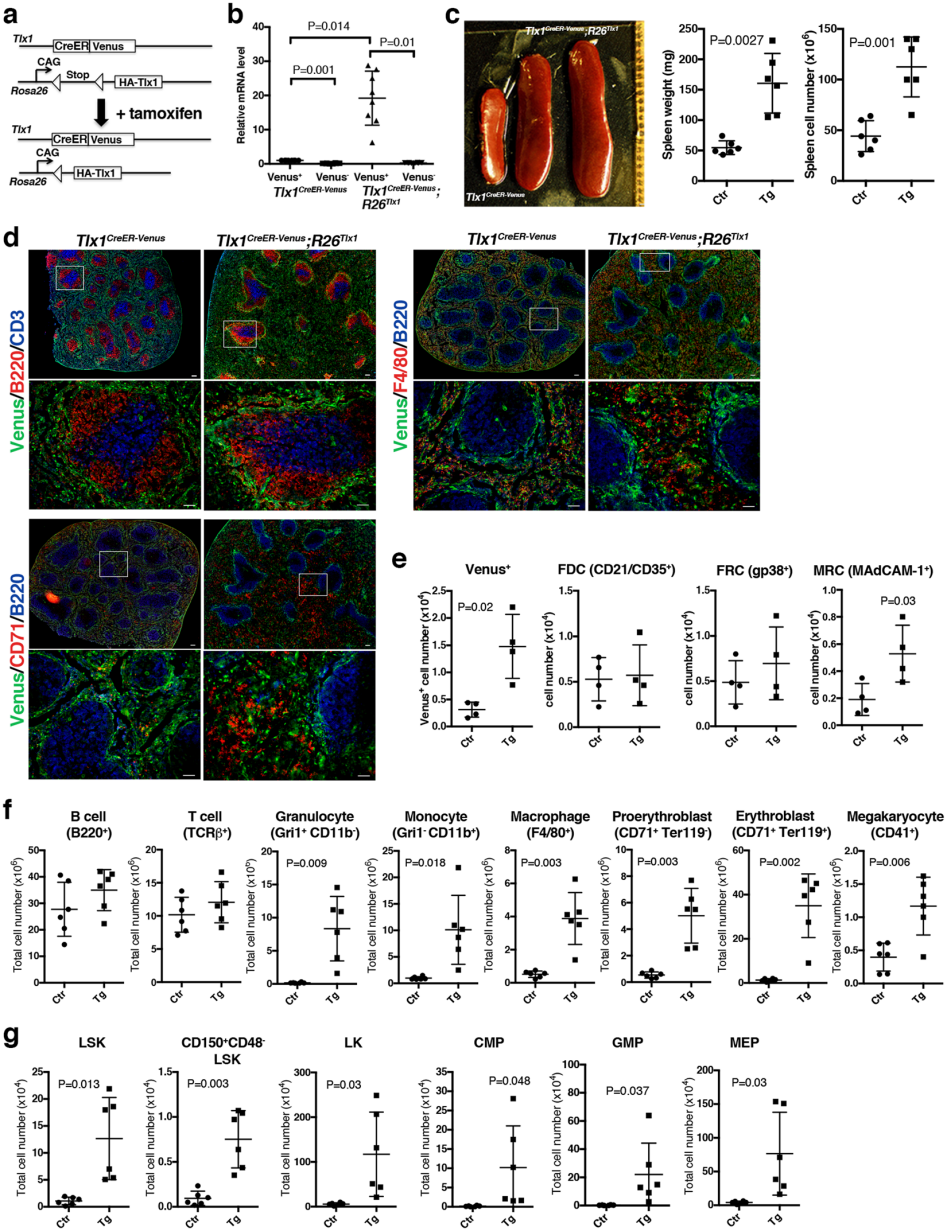
Overexpression of Tlx1 *in situ* in the spleen induces EMH. (**a**) Schematic presentation of experimental strategy to overexpress *Tlx1* in *Tlx1*^+^ cells. *Tlx1*^*CreER-Venus*^ littermate controls and *Tlx1*^*CreER-Venus*^*; R26*^*Tlx1*^ mice (4-week-old) were treated with tamoxifen, and analyzed 24 hours (**b**) and 8 weeks after the final treatment (**c**–**g**). Data were pooled from 2–4 independent experiments. (**b)** Expression levels of the sum of exogenous *Tlx1* transgene and endogenous *Tlx1* mRNA in the Venus^+^ cells or Venus^−^ cells gated on CD45^−^ Ter119^−^ CD31^−^ stromal cells from the spleen of *Tlx1* ^*CreER-Venus*^ controls (Ctr) and *Tlx1*^*CreER-Venus*^*; R26*^*Tlx1*^ mice (Tg) with tamoxifen treatment. Data were normalized to β*-actin* and the level of mRNA transcripts in Venus^+^ cells of *Tlx1*^*CreER-Venus*^ controls was arbitrarily set to 1. (mean ± SD, n = 8). (**c)** Gross appearance (left), the weight (middle) and cell numbers (right) of the spleen from tamoxifen-treated *Tlx1*^*CreER-Venus*^*; R26*^*Tlx1*^ mice (Tg), compared to the spleen from tamoxifen-treated *Tlx1*^*CreER-Venus*^ littermates (Ctr). (mean ± SD, n = 7). (**d)** Immunohistochemical analysis of the spleen from tamoxifen-treated *Tlx1*^*CreER-Venus*^ controls and *Tlx1*^*CreER-Venus*^*; R26*^*Tlx1*^ mice. Tissue sections were stained with the indicated antibody combinations. Lower magnification images are indicated by an inserted rectangle in the upper images. Scale bars indicate 100 μm (upper) and 50 μm (lower). (n = 3). (**e)** Total cell numbers of the indicated stromal cell populations from the spleen of tamoxifen-treated *Tlx1*^*CreER-Venus*^ controls (Ctr) and *Tlx1*^*CreER-Venus*^*; R26*^*Tlx1*^ mice (Tg). (mean ± SD; n = 4). (**f)** Total numbers of the indicated mature hematopoietic cell populations from the spleen of tamoxifen-treated *Tlx1*^*CreER-Venus*^ controls (Ctr) and tamoxifen-treated *Tlx1*^*CreER-Venus*^*; R26*^*Tlx1*^ mice (Tg). (mean ± SD; n = 6). (**g)** Total numbers of the indicated hematopoietic stem/progenitor cell populations from the spleen of tamoxifen-treated *Tlx1*^*CreER-Venus*^ controls (Ctr) and tamoxifen-treated *Tlx1*^*CreER-Venus*^*; R26*^*Tlx1*^ mice (Tg). (mean ± SD; n = 6).

As we have observed that the intensity of Venus fluorescence in the splenic mesenchymal cells gradually decreased after birth in *Tlx1*^*CreER-Venus*^ mice (data will be described elsewhere), we predicted that reduction in Tlx1 expression might inversely correlates with the hematopoietic activity of the spleen, which functions as a hematopoietic organ during the perinatal period until the bone marrow becomes a fully functional hematopoietic niche. To address this prediction, we sought to overexpress exogenous Tlx1 *in situ* in postnatal Tlx1-expressing cells by crossing *Tlx1*^*CreER-Venus*^ mice with *R26*^*Tlx1*^ mice harboring a novel *R26* mutant allele in which a HA epitope-tagged *Tlx1* cDNA is inserted 3′ of the *CAG* promoter with the *lox*P-flanked stop cassette (Fig. 2a). In the *Tlx1*^*CreER-Venus*^*; R26*^*Tlx1*^ mice, tamoxifen treatment resulted in about a 20-fold increase in total *Tlx1* mRNA expression, including endogenous *Tlx1* mRNA, in Venus^+^ cells, as compared to Venus^+^ cells from non-treated controls and from tamoxifen-treated *Tlx1*^*CreER-Venus*^ mice (Fig. 2b). When we examined the effect of Tlx1 overexpression *in situ* in the spleen 4 weeks after tamoxifen treatment, the size, weight and cell numbers of the spleen were not significantly altered (Fig. S2a); however, marked splenomegaly with significantly increased splenic weight was observed in *Tlx1*^*CreER-Venus*^*; R26*^*Tlx1*^ mice 8 weeks after tamoxifen treatment, as compared with tamoxifen-treated *Tlx1*^*CreER-Venus*^ mice (Fig. 2c). Immunohistochemical analyses revealed that Tlx1 overexpression causes expansion of the red pulp area with an apparently intact white pulp and accumulation of Venus^high^ cells in the perifollicular area (Fig. 2d). In the enlarged red pulp area of tamoxifen-treated *Tlx1*^*CreER-Venus*^*; R26*^*Tlx1*^ spleen, CD71^+^ erythroid cells as well as F4/80^+^ macrophages were increased, as compared to the tamoxifen-treated *Tlx1*^*CreER-Venus*^ spleen (Fig. 2d). Flow cytometric analyses of splenic stromal cell numbers also revealed an increase in Venus^+^ cells as well as MRCs that were localized adjacent to the Venus^+^ cells in the perifollicular area with no significant alteration in the stromal cell components of the white pulp, including FRCs, and FDCs, upon Tlx1 overexpression (Fig. 2e).

The hematopoietic cell compartment of the spleen also gradually altered from 4 weeks to 8 weeks upon Tlx1 overexpression. The numbers of erythrocytes, granulocytes and macrophages as well as granulocyte/monocyte progenitors (GMPs: Lin^−^Sca-1^−^c-Kit^+^FcγRII/III^high^ CD34^high^) were significantly, increased in *Tlx1*^*CreER-Venus*^*; R26*^*Tlx1*^ mice 4 weeks after tamoxifen treatment, as compared to those in controls (Fig. S2b,c), while the numbers of non-lineage-restricted compartments, including LSK (Lin^−^Sca-1^+^c-Kit^+^), CD150^+^CD48^−^LSK enriched in HSCs and subsequent lineage-committed LK (Lin^−^Sca-1^−^c-Kit^+^) cells as well as common myeloid progenitors (CMPs; Lin^−^Sca-1^−^c-Kit^+^FcγRII/III^int^CD34^high^) and megakaryocyte/erythroid progenitors (MEPs; Lin^−^Sca-1^−^c-Kit^+^FcγRII/III^−^ CD34^low^) were not significantly elevated upon Tlx1 overexpression at this time point (Fig. S2b). However, 8 weeks after tamoxifen treatment, the numbers of myeloid-lineage cells, including granulocytes, monocytes and macrophages, were significantly increased upon Tlx1 overexpression (Fig. 2f). Furthermore, a significant increase in the number of both erythroid-lineage cells and megakaryocytes was observed in tamoxifen-treated *Tlx1*^*CreER-Venus*^*; R26*^*Tlx1*^ mice (Fig. 2f). Since these multi-lineage increments in the mature hematopoietic cells suggest the possibility of EMH in the spleen, we further examined non-lineage-restricted and lineage-restricted immature hematopoietic cell compartments. The numbers of cells in the immature hematopoietic compartments, including LSK cells as well as CD150^+^CD48^−^LSK cells were significantly elevated upon Tlx1 overexpression, in association with LK cells, including common myeloid progenitors CMPs, GMPs and MEPs (Fig. 2g). These findings indicate that a high level of prolonged Tlx1 expression *in situ* in the spleen mesenchymal cells gradually alters the spleen to become an active site for EMH.

### High levels of Tlx1 expression in the spleen alter BM hematopoiesis

**Figure 3:**
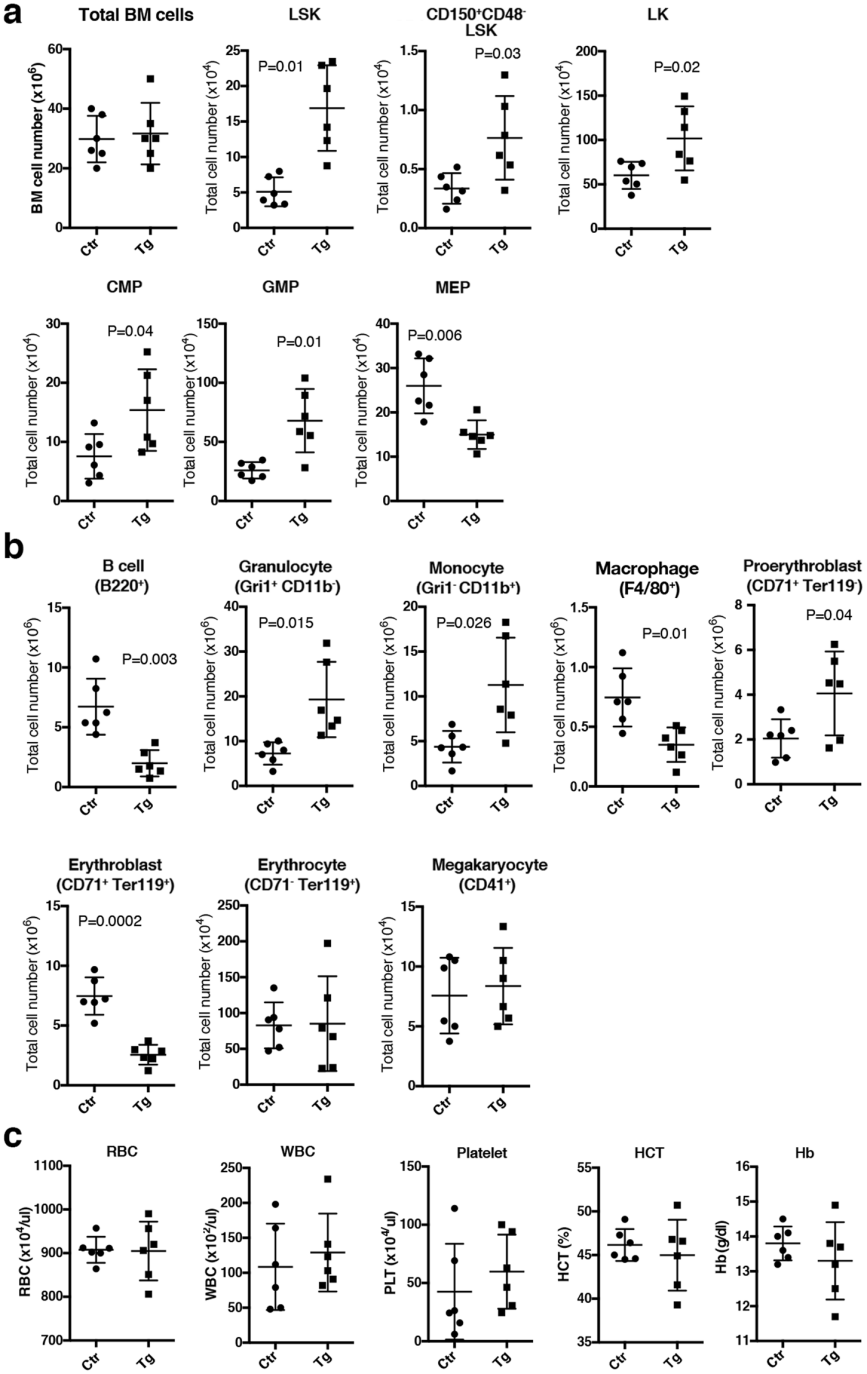
Effect of Tlx1 overexpression *in situ* in the spleen on BM hematopoiesis. *Tlx1*^*CreER-Venus*^ littermate controls and *Tlx1*^*CreER-Venus*^*; R26*^*Tlx1*^ mice (4-week-old) were treated with tamoxifen and hematopoietic cell populations in the BM and the peripheral blood were analyzed 8 weeks after the final treatment. Data were pooled from 4 independent experiments. (**a)** Total numbers of the indicated hematopoietic stem/progenitor cell populations in the BM of tamoxifen-treated *Tlx1*^*CreER-Venus*^ controls (Ctr) and tamoxifen-treated *Tlx1*^*CreER-Venus*^*; R26*^*Tlx1*^ mice (Tg). (mean ± SD; n = 6). (**b)** Total numbers of the indicated mature hematopoietic cell populations in the BM of tamoxifen-treated *Tlx1*^*CreER-Venus*^ controls (Ctr) and tamoxifen-treated *Tlx1*^*CreER-Venus*^*; R26*^*Tlx1*^ mice (Tg). (mean ± SD; n = 6). (**c)** Hematological indices of peripheral blood of tamoxifen-treated *Tlx1*^*CreER-Venus*^ controls (Ctr) and tamoxifen-treated *Tlx1*^*CreER-Venus*^*; R26*^*Tlx1*^ mice (Tg). RBC indicates red blood cell count; WBC, white blood cell count; Platelets, platelet cell count; HCT, hematocrit; Hb, hemoglobin. (mean ± SD; n = 6).

We next examined the effect of Tlx1-mediated splenic EMH on BM hematopoiesis. No significant alterations in mature or progenitor cell compartments was observed in the BM of *Tlx1*^*CreER-Venus*^*; R26*^*Tlx1*^ mice 4 weeks after tamoxifen treatment (Fig. S2d,e). However, a significant alteration in BM hematopoiesis was observed 8 weeks after Tlx1 overexpression. The numbers of LSK and CD150^+^CD48^−^LSK cells were modestly but significantly elevated in tamoxifen-treated *Tlx1*^*CreER-Venus*^*; R26*^*Tlx1*^ mice, as compared to the controls (Fig. 3a). In the LK cell compartment, the numbers of CMPs and GMPs were significantly increased but, in contrast, MEP cell numbers were significantly decreased as compared to the controls (Fig. 3a). Reflecting the alteration in the lineage-committed progenitor cells, the numbers of granulocytes, monocytes and macrophages were significantly increased, whereas the CD71^+^Ter119^+^ erythroblasts were significantly reduced, accompanied by a significant elevation of CD71^+^Ter119^−^ proerythroblasts, in tamoxifen-treated *Tlx1*^*CreER-Venus*^*; R26*^*Tlx1*^ mice as compared to the controls (Fig. 3b). In addition, B-lineage cell numbers in the BM was also significantly reduced upon Tlx1 overexpression, suggesting myeloid-biased hematopoiesis in the BM (Fig. 3b). Despite of these dynamic alterations in BM hematopoiesis, the number of red blood cells (RBCs) in the peripheral blood was not significantly altered upon Tlx1 overexpression, with apparently normal hematocrit values and hemoglobin concentrations, suggesting that erythropoiesis both in the spleen and the BM maintains a balance of systemic RBC numbers for a strict regulation of oxygen supply (Fig. 3c). The numbers of total white blood cells (WBCs) and platelets were modestly, albeit not significantly, increased upon Tlx1 overexpression (Fig. 3c). These findings thus indicate that EMH in the spleen caused by a high level of Tlx1 expression indirectly affects BM hematopoiesis, although we could not rule out the possibility that rare Tlx1-expressing cells, undetectable by the flow cytometry analysis, are present in the BM and directly participate in BM hematopoiesis.

### High levels of Tlx1 expression *in situ* promote circulation of HSPCs in the periphery and their recruitment to and proliferation in the spleen

**Figure 4:**
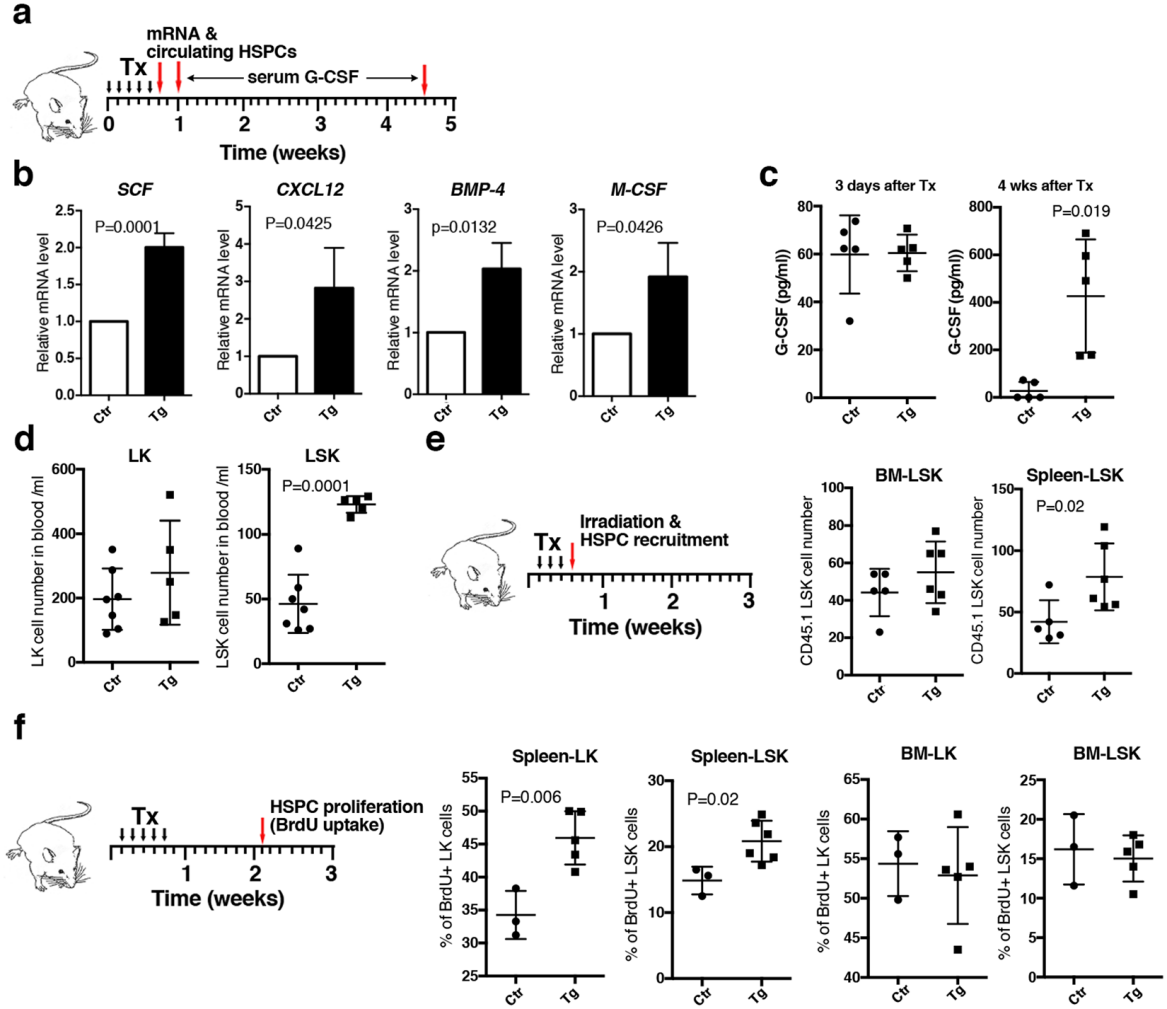
Early phase effects of Tlx1 overexpression *in situ* in the spleen on hematopoietic factor expression and behavior of HSPCs. (**a**) Schematic of the experimental strategy represents time points of the analyses for hematopoietic factor mRNA and serum G-CSF as well as peripheral blood HSPCs. Data were pooled from 2–3 independent experiments. (**b**) Expression of the indicated hematopoietic factor mRNA in Venus^+^ cells of the spleen from tamoxifen-treated *Tlx1*^*CreER-Venus*^ controls (Ctr) and tamoxifen-treated *Tlx1*^*CreER-Venus*^*; R26*^*Tlx1*^ mice (Tg). (mean ± SD, n = 3). The relative mRNA levels were normalized to *β-actin* and the level of mRNA transcripts in Venus^+^ cells of tamoxifen-treated *Tlx1*^*CreER-Venus*^ controls was arbitrarily set to 1. (**c**) Serum G-CSF concentration of tamoxifen-treated *Tlx1*^*CreER-Venus*^ controls (Ctr) and tamoxifen-treated *Tlx1*^*CreER-Venus*^*; R26*^*Tlx1*^ mice (Tg). (mean ± SD, n = 5). (**d**) Total numbers of the indicated HSPC populations in the peripheral blood of tamoxifen-treated *Tlx1*^*CreER-Venus*^ controls (Ctr) and tamoxifen-treated *Tlx1*^*CreER-Venus*^*; R26*^*Tlx1*^ mice (Tg). (mean ± SD, n = 5–7). (**e**) Total cell numbers of transplanted CD45.1 LSK cells in the spleen and BM of tamoxifen-treated *Tlx1*^*CreER-Venus*^ controls (Ctr) and tamoxifen-treated *Tlx1*^*CreER-Venus*^*; R26*^*Tlx1*^ mice (Tg). (mean ± SD, n = 5–6). (**f**) The frequency of BrdU-incorporated LSK and LK cells in the spleen and BM of tamoxifen-treated *Tlx1*^*CreER-Venus*^ littermate controls (Ctr) and tamoxifen-treated *Tlx1*^*CreER-Venus*^*; R26*^*Tlx1*^ mice (Tg). (mean ± SD, n = 3–5).

To understand the mechanism by which a high level of Tlx1 expression in splenic mesenchymal cells induces EMH, early stage alterations in splenic hematopoiesis with respect to hematopoietic factor expression as well as the dynamics of HSPCs were examined. We first analyzed hematopoietic factor expression in Venus^+^ cells and mobilization of HSPCs into the peripheral blood 24 hours after inducing Tlx1 overexpression (Fig. 4a, upper cartoon). We found a significant elevation in gene transcripts of not only hematopoietic niche factors, *CXCL12* and *SCF*, that were highly enriched in steady-state Venus^+^ cells, but also bone morphologic factor-4 (*BMP-4*) and macrophage colony-stimulating factor (*M-CSF*) that have been reported to participate in splenic erythropoiesis^26,27^ and differentiation of monocytes^28^, respectively (Fig. 4b). In contrast, serum levels of G-CSF, which induces HSPC mobilization from the BM^29,30^, were not significantly altered after 3 days of Tlx1 overexpression, although we did find a significant elevation in G-CSF serum levels 4 weeks post Tlx1 overexpression (Fig. 4c). Nevertheless, LSK cells, but not LK cells, were significantly increased in the peripheral blood 24 hours after inducing Tlx1 overexpression (Fig. 4d), suggesting that factors other than G-CSF enhance mobilization of HSPCs from the BM to the periphery.

We next analyzed the recruitment of such circulating HSPCs to the spleen by intravenously injecting CD45.1^+^ LSK cells into lethally-irradiated CD45.2^+^ host mice. While there was no significant alteration in transferred LSK homing to the BM after Tlx1 overexpression, a significantly higher number of transferred LSK cells was detected in the spleen (Fig. 4e), indicating a preferential recruitment of circulating HSPCs to the spleen with high Tlx1 expression in the stromal cell compartment. To address whether HSPCs in the spleen are actively cycling or quiescent under these conditions, we performed a 1-hour-BrdU pulse at 10 days after Tlx1 overexpression. As shown in Fig. 4f, Tlx1 overexpression significantly increased the numbers of BrdU-incorporated LSK cells as well as LK cells without affecting proliferation of BM LSK and LK cells. Taken together, these findings indicate that a high level of Tlx1 expression in the spleen induces HSPCs mobilization into the periphery and their recruitment to the spleen, where they proliferate and respond to various hematopoietic factors produced by niche cells, thus leading to splenic EMH.

### High levels of Tlx1 expression *in situ* create an HSPC niche for EMH in the spleen

**Figure 5:**
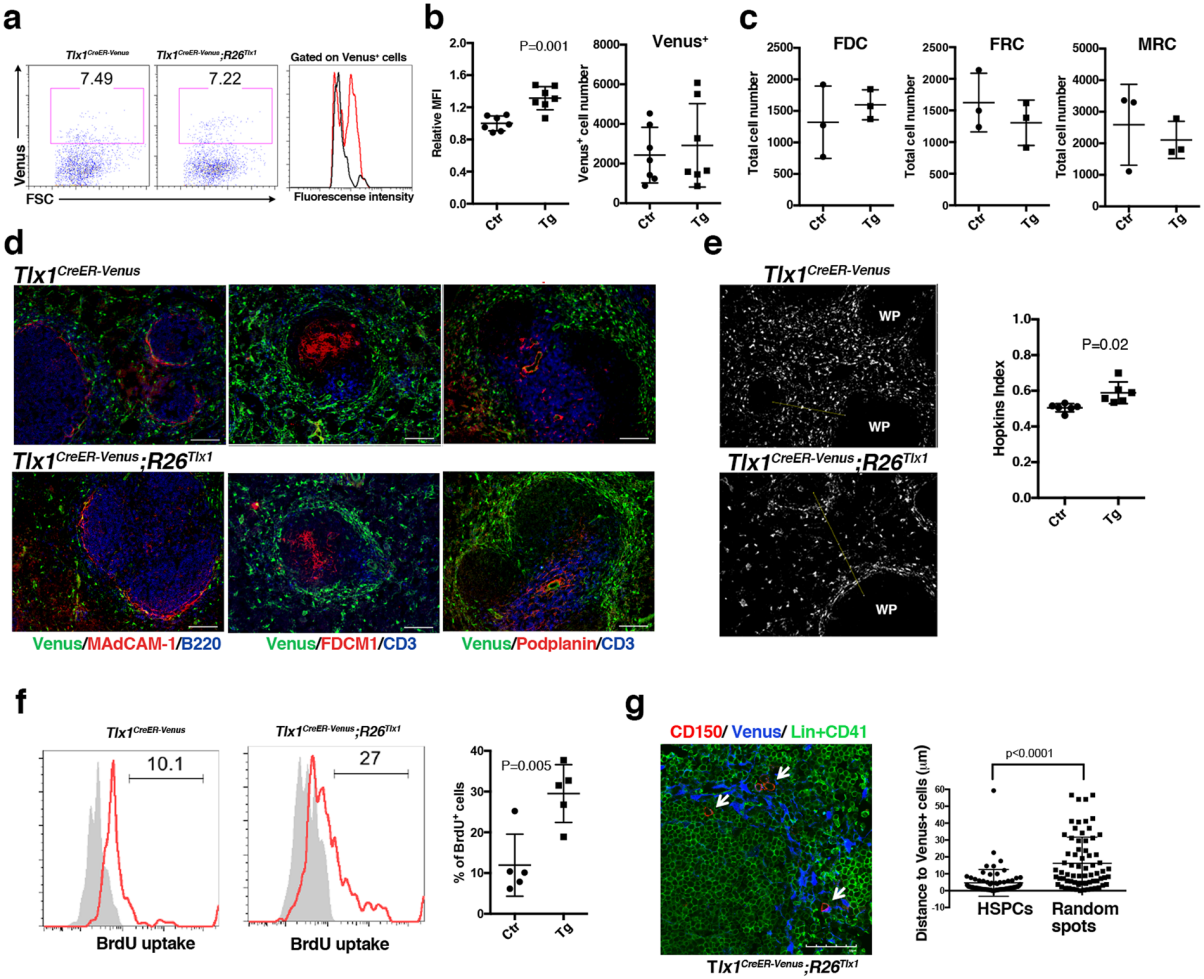
Tlx1-expressing cells function as a HSPC niche for EMH in the spleen. *Tlx1*^*CreER-Venus*^ littermate controls and *Tlx1*^*CreER-Venus*^*; R26*^*Tlx1*^ mice (4-week-old) were treated with tamoxifen and stromal cell populations in the spleen were analyzed 24 hours after the final treatment. Data were pooled from 2–4 independent experiments. (**a)** Representative flow cytometric profiles of CD45^−^ Ter119^−^ CD31^−^ stromal cells from the spleen of tamoxifen-treated *Tlx1*^*CreER-Venus*^ controls (left) and *Tlx1*^*CreER-Venus*^*; R26*^*Tlx1*^ mice (middle). Gates used to identify Venus^+^ cell population are outlined, and numbers above the outlined areas indicate percent events in each gate. A histogram (right) represents the intensity of Venus fluorescence of Venus^+^ cells from tamoxifen-treated *Tlx1*^*CreER-Venus*^ controls (black line) and *Tlx1*^*CreER-Venus*^*; R26*^*Tlx1*^ mice (red line). (**b)** Graphs represent the MFI of Venus fluorescence and the numbers of Venus^+^ cells from the spleen of tamoxifen-treated *Tlx1*^*CreER-Venus*^ littermate controls (Ctr) and tamoxifen-treated *Tlx1*^*CreER-Venus*^*; R26*^*Tlx1*^ mice (Tg). The relative MFI of Venus^+^ cells in tamoxifen-treated *Tlx1*^*CreER-Venus*^ controls was arbitrarily set to 1. (mean ± SD; n = 7). (**c)** Total numbers of the indicated stromal cell populations from the spleen of tamoxifen-treated *Tlx1*^*CreER-Venus*^ controls (Ctr) and tamoxifen-treated *Tlx1*^*CreER-Venus*^*; R26*^*Tlx1*^ mice (Tg). (mean ± SD; n = 3). (**d)** Immunohistochemical analysis of the spleen of tamoxifen-treated *Tlx1*^*CreRE-Venus*^ littermate controls and *Tlx1*^*CreER-Venus*^*; R26*^*Tlx1*^ mice. Tissue sections were stained with the indicated antibody combinations. Scale bars indicate 100 μm. (n = 3). (**e)** Clustering analysis of Venus^+^ cells in the spleen upon Tlx1 overexpression. Representative digital images of Venus^+^ cells (white dots) in the spleen sections from the indicated mice. WP, the white pulp area. The right graph represents the Hopkins index of Venus^+^ cells in the spleen of tamoxifen-treated *Tlx1*^*CrER-Venus*^ controls (Ctr) and *Tlx1*^*CreER-Venus*^*; R26*^*Tlx1*^ mice (Tg). (n = 6). (**f)** BrdU incorporation analysis of Venus^+^ cells upon Tlx1 overexpression. Representative flow cytometric histograms of Venus^+^ cells of the indicated mice stained with anti-BrdU antibody (red line) and isotype control antibody (shaded line). The right graph represents the percentage of BrdU^+^ cells among total Venus^+^ cells in the spleen of tamoxifen-treated *Tlx1*^*CreER-Venus*^ controls (Ctr) and *Tlx1*^*CreER-Venus*^*; R26*^*Tlx1*^ mice (Tg). (mean ± SD, n = 5). (**g)** Distance of Venus^+^ cells from CD150^+^CD41^−^Lin^−^ HSPCs. Tissue sections of the spleen from tamoxifen-treated *Tlx1*^*CreER-Venus*^*; R26*^*Tlx1*^ mice were stained with the indicated antibody combinations. White arrows indicate HSPCs. The right graph represents the distance of Venus^+^ cells from CD150^+^CD41^−^Lin^−^ HSPCs or randomly generated spots. (n = 93 from 3 mice).

To gain insight into the Tlx1-mediated enhancement of HSPC supportive activity of the spleen, we next examined alterations in the stromal cell compartment at an early time point after Tlx1 overexpression (24 hours after the final tamoxifen treatment). We first found a remarkable increase in Venus fluorescent intensity in the CD45^−^Ter119^−^ non-hematopoietic stromal cell compartment upon Tlx1 overexpression (Fig. 5a), which is consistent with a previous finding that Tlx1 binds its own promoter to positively regulate its transcription^19^. Indeed, the mean Venus fluorescence intensity (MFI) was significantly increased upon Tlx1 overexpression (Fig. 5b). Although total numbers of Venus^+^ cells as well as other stromal cells, including FRCs, FDCs and MRCs, were not significantly altered upon Tlx1 overexpression (Fig. 5b,c), immunohistochemical observations revealed an increased tendency for Venus^+^ cells to accumulate around the perifollicular region surrounding the white pulp (Fig. 5d). In the control spleen, Venus^+^ cells appeared to be randomly scattered in the entire red pulp; however, upon Tlx1 overexpression, these cells appeared to preferentially localize at the perifollicular region. To gain a quantitative insight into the distribution of Venus^+^ cells in the red pulp, we applied the Hopkins index to digitally reconstructed randomly selected images, as described in the Methods. Consistent with our visual inspection, Venus^+^ cells in the red pulp upon Tlx1 overexpression had a Hopkins index that was significantly higher (0.59 ± 0.03) than that expected for a fully random distribution (0.5), whereas the Hopkins index of Venus^+^ cells in the controls was 0.50 ± 0.01 (Fig. 5e), supporting the hypothesis that a high level Tlx1 expression induces biased localization of Venus^+^ cells near the perifollicular area. Furthermore, BrdU incorporation by Venus^+^ cells was significantly increased upon Tlx1 overexpression, indicating that a high level of Tlx1 expression also promotes cell proliferation (Fig. 5f). When we analyzed the localization of CD150^+^CD41^−^Lin^−^ HSPCs in relation to Venus^+^ cells, we found that they were localized close to the Venus^+^ cells that accumulate in the perifollicular area of the red pulp upon Tlx1 overexpression (Fig. 5g, white arrows in right photo). Indeed, CD150^+^CD41^−^Lin^−^ cells were significantly more likely to be close to Venus^+^ cells (4.59 ± 0.94 μm) than randomly localized (16.21 ± 1.83 μm) (Fig. 5g), suggesting that Venus^+^ cells indeed act as a niche for HSPCs in the spleen. Taken together, these findings suggest that a high level Tlx1 expression creates a HSPC niche in the perifollicular region of the spleen to support EMH.

### Requirement for a high level of Tlx1 expression in LPS-induced EMH

**Figure 6:**
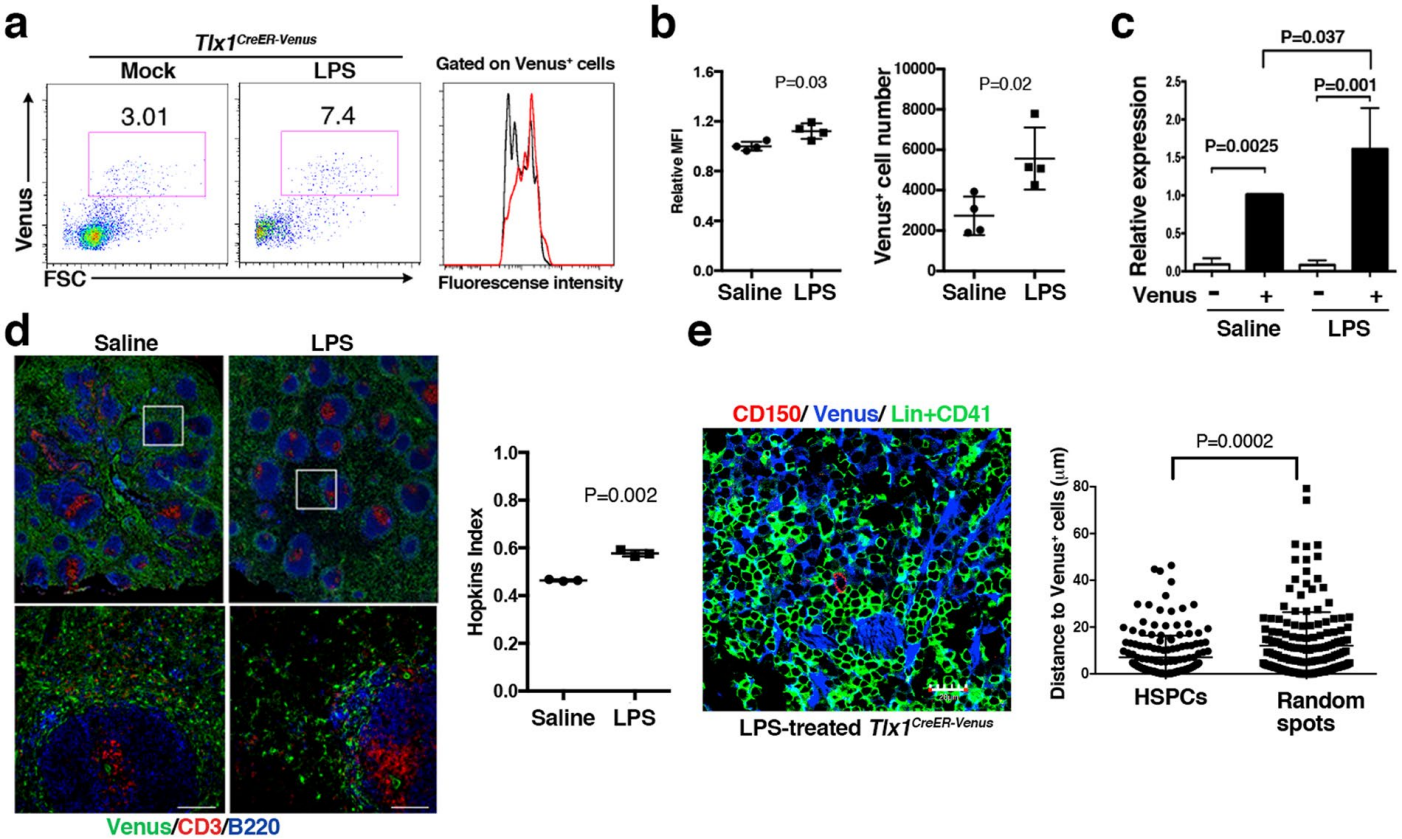
Alterations in Tlx1-expressing cells upon LPS treatment. *Tlx1*^*CreER-Venus*^ mice (4-week-old) were treated with LPS for 3 consecutive days, and stromal cell populations in the spleen were analyzed 24 hours after the final treatment. (**a)** Representative flow cytometric profiles of CD45^−^ Ter119^−^ CD31^−^ stromal cells in the spleen of *Tlx1*^*CreER-Venus*^ mice treated with LPS (middle) or endotoxin-free saline (left). Gates used to identify Venus^+^ cell populations are outlined and numbers above the outlined areas indicate percent events in each gate. The histogram represents the intensity of fluorescence of Venus^+^ cells from *Tlx1*^*CreER-Venus*^ mice treated with LPS (red line) or saline (black line). (**b)** The MFI and total cell numbers of Venus^+^ cells of *Tlx1*^*CreER-Venus*^ mice treated with LPS or saline. The relative MFI of Venus^+^ cells in saline-treated *Tlx1*^*CreER-Venus*^ controls was arbitrarily set to 1. (n = 4). (**c)** Endogenous *Tlx1* mRNA expression by Venus^−^ and Venus^+^ cells of *Tlx1*^*CreER-Venus*^ mice treated with LPS or saline. The relative mRNA levels were normalized to β*-actin* and the level of mRNA transcripts in Venus^−^ cells of saline-treated *Tlx1*^*CreER*^ controls was arbitrarily set to 1. (n = 3). (**d)** Representative tissue section images of spleen from saline- or LPS-treated *Tlx1*^*CreER-Venus*^ mice. Tissue sections were stained with the indicated antibody combinations (upper photos). The inserted rectangle is shown at a higher magnification in the lower images. Scale bars indicate 100 μm. The right graph represents the Hopkins index of Venus^+^ cells in the spleen of saline- or LPS-treated *Tlx1*^*CreER-Venus*^ mice. (n = 3). (**e)** Distance of Venus^+^ cells from CD150^+^ CD41^−^ Lin^−^ HSPCs (n = 172 from 3 mice). Tissue sections from the spleen of LPS-treated *Tlx1*^*CreER-Venus*^ mice were stained with the indicated antibody combinations. The right graph represents the distance of Venus^+^ cells from CD150^+^ CD41^−^ Lin^−^ HSPCs or randomly generated spots.

To address whether these observations can be extrapolated to a more biologically relevant, albeit pathophysiological form of EMH^30^, we examined the involvement of Tlx1-expresssing mesenchymal cells in LPS-induced EMH. To create this model, we administrated LPS or endotoxin-free saline intraperitoneally into *Tlx1*^*CreER-Venus*^ mice for 3 consecutive days and then examined EMH in the spleen 24 hours after the final treatment. As observed upon Tlx1 overexpression *in situ* in the spleen (Fig. 5a), the proportion of Venus^high^ cells was profoundly increased (Fig. 6a), with a significant increment in MFI and cell numbers of Venus^+^ cells upon LPS treatment, respectively (Fig. 6b). This observation was further confirmed at the endogenous *Tlx1* gene transcript level, in which *Tlx1* mRNA expression was significantly enhanced in Venus^+^ cells by LPS treatment (Fig. 6c). Immunohistochemical analyses also revealed a tendency for Venus^+^ cell accumulation in the perifollicular area surrounding the white pulp after LPS treatment (Fig. 6d, left panels), and the Hopkins index indicated a nonrandom distribution of Venus^+^ cells after LPS treatment (0.58 ± 0.01 versus 0.46 ± 0.003, Fig. 6d, right graph). Furthermore, CD150^+^CD41^−^Lin^−^ cells were again detectable close to Venus^+^ cells that accumulate to the perifollicular area of the red pulp (Fig. 6e, left), and these cells showed a significant association with Venus^+^ cells (7.10 ± 0.01 μm) rather than a random localization (12.01 ± 1.09 μm) (Fig. 6e right). Thus, LPS-induced changes in Tlx1-expressing cells appears to partly recapitulate those induced by Tlx1-overexpression *in situ* in the spleen.

**Figure 7:**
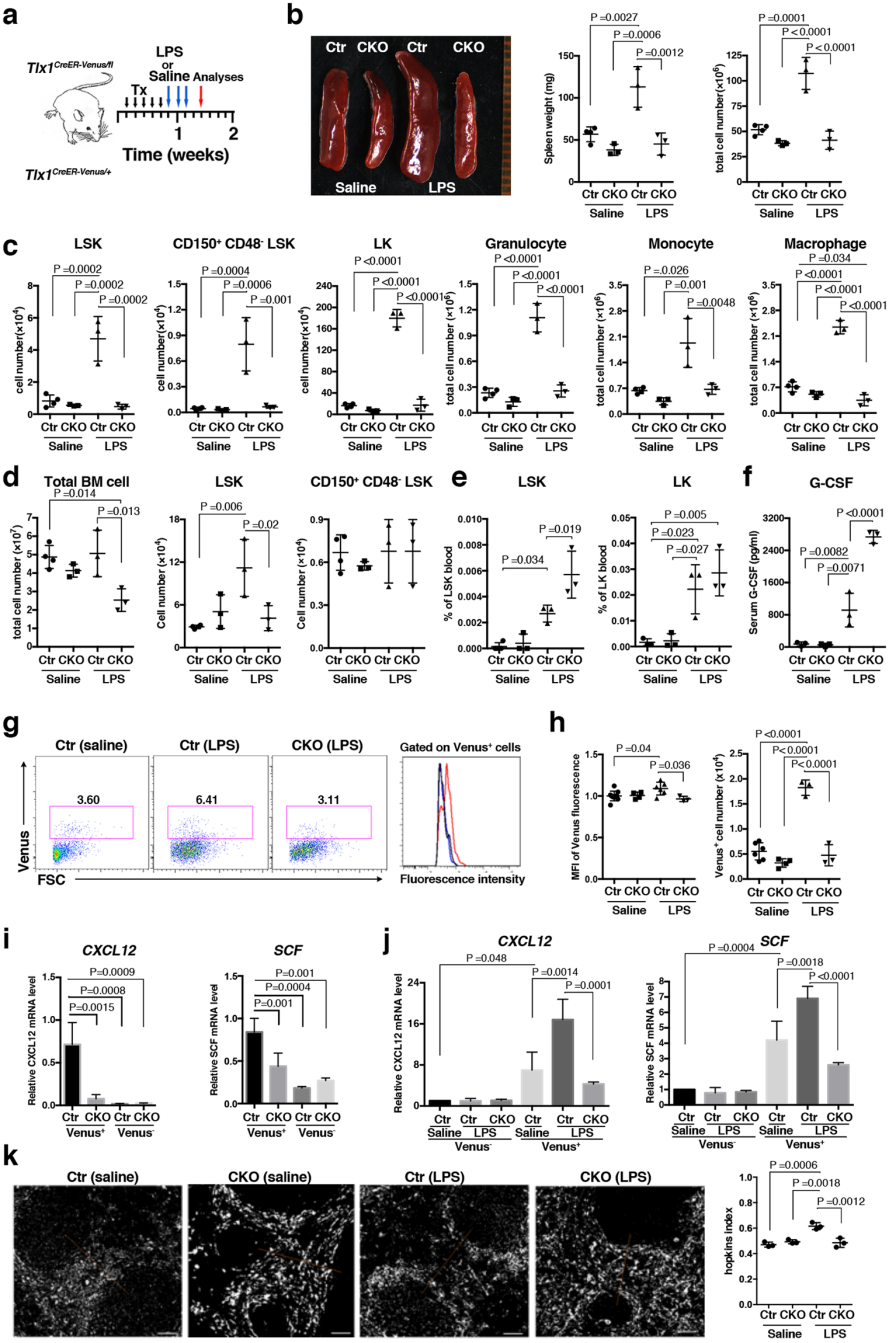
Tlx1 is required for LPS-induced EMH in the spleen. (**a**) Schematic of the experimental strategy including time points of the analysis. *Tlx1*^*CreER-Venus*/+^ littermate control mice (Ctr) and *Tlx1*^*CreER-Venus/fl*^ mice (CKO) treated with tamoxifen for 5 consecutive days were administered LPS or endotoxin-free saline for an additional 3 consecutive days and then analyzed 48 hours after the final treatment. Data were pooled from at least 2 independent experiments. (**b)** Gross appearance (left), weight (middle) and total cell number (right) of the spleen from Ctr and CKO mice treated with LPS or saline. (n = 3–4). (**c)** Total cell numbers of the indicated hematopoietic cell populations from the spleen of Ctr and CKO mice treated with saline or LPS. The gating strategy was same as in Fig. 3a. (n = 3–4). (**d**) Total cell numbers of the indicated hematopoietic stem/ progenitor cell populations in the BM of Ctr and CKO mice treated with saline or LPS. The gating strategy was same as in Fig. 2f,g. (n = 3–4). (**e)** The percentage of the indicated hematopoietic stem/ progenitor cell populations in the peripheral blood of Ctr and CKO mice treated with saline or LPS. (n = 3). (**f)** Serum G-CSF concentration in Ctr and CKO mice treated with saline or LPS. (n = 3). (**g)** Representative flow cytometric profiles of CD45^−^ Ter119^−^ CD31^−^ stromal cells from the spleen of Ctr and CKO mice treated with saline or LPS. Gates used to identify Venus^+^ cell populations are outlined and numbers above the outlined areas indicate percent events in each gate. The histogram represents the intensity of fluorescence of Venus^+^ cells from Ctr mice treated with LPS (red line) or saline (black line), and CKO mice treated with LPS (blue). (**h)** The MFI and total cell numbers of Venus^+^ cells of Ctr and CKO mice treated with saline or LPS. The relative MFI of Venus^+^ cells in the saline-treated Ctr was arbitrarily set to 1. (n = 3–6). (**i)** Expression of *CXCL12* and *SCF* mRNA in Venus^+^ and Venus^−^ cells among CD45^−^ Ter119^−^ CD31^−^ splenic stroma cells from Ctr and CKO mice 24 hours after tamoxifen treatment. Data were normalized to β*-actin* and the level of mRNA transcripts in Venus^+^ cells of Ctr mice was arbitrarily set to 1. (n = 3). (**j)** Expression of *CXCL12* and *SCF* mRNA in splenic Venus^+^ and Venus^−^ cells of saline-treated Ctr mice, LPS-treated Ctr and LPS-treated CKO mice. Data were normalized to *β-actin* and the level of mRNA transcripts in Venus^−^ cells of saline-treated Ctr mice was arbitrarily set to 1. (n = 3). (**k)** Clustering analysis of Venus^+^ cells in the spleen from Ctr and CKO mice treated with LPS or saline. Representative digital images of Venus^+^ cells (white dots) in the spleen sections from the indicated mice. The right graph represents the Hopkins index of Venus^+^ cells in the spleen of Ctr and CKO mice treated with saline or LPS. (n = 3).

To assess whether Tlx1 expression functionally participates in LPS-induced EMH, we generated an LPS-induced EMH model in *Tlx1*^*fl*^ mice harboring the *Tlx1*^*CreER-Venus*^ allele (*Tlx1*^*CreER-Venus/fl*^) to conditionally delete the *Tlx1* coding exon upon tamoxifen treatment (Fig. 7a). In this model the *Tlx1* gene is selectively deleted in Venus^+^ cells without the loss of Venus^+^ cells upon tamoxifen treatment (Fig. S3). The loss of Tlx1 itself did not significantly affect either size or weight of the spleen; however, it almost completely abolished LPS-induced spleen enlargement (Fig. 7b). The increase in spleen cell numbers normally seen after LPS treatment was also abolished by Tlx1 loss. This was associated with a significant reduction in LPS-induced elevation of LSK and CD150^+^CD48^−^ LSK cell numbers, as well as of LK cells, to the levels seen in the saline-treated controls (Fig. 7c). In line with this, the elevated numbers of mature granulocytes, monocytes and macrophages normally induced by LPS treatment was similarly reduced to the level of saline-treated controls by the loss of Tlx1 (Fig. 7c). To our surprise, although Tlx1 loss itself did not significantly affect BM hematopoiesis, the loss of Tlx1 significantly reduced the total cell number of BM cells as well as the elevation in numbers of LSK cells in the BM upon LPS treatment (Fig. 7d). In contrast, the elevation in the frequencies of both LSK and LK cells in the peripheral blood induced by LPS was significantly enhanced by the loss of Tlx1 (Fig. 7e). LPS-induced mobilization of HSPCs from the BM to the periphery is dependent on the release of G-CSF^29,30^. LPS treatment itself significantly augmented serum G-CSF levels, and this was further augmented by Tlx1 loss (Fig. 7f), although Tlx1 loss itself did not affect serum G-CSF levels. Next, we examined the LPS-induced changes in Tlx1-expressing cells upon Tlx1 loss and found that both the LPS-induced increase in Venus expression levels and in Venus^+^ cell numbers were completely eliminated by Tlx1 loss (Fig. 7g,h). Furthermore, Tlx1 loss significantly reduced both basal and LPS-induced *CXCL12* and *SCF* mRNA levels in Venus^+^ cells, with no alteration of these levels in Venus^−^ cells (Fig. 7i,j). In addition, perifollicular accumulation of Venus^+^ cells upon LPS treatment was eliminated by Tlx1 loss (0.61 ± 0.03 in controls versus 0.48 ± 0.04 in Tlx1-deficient cells, Fig. 7i), although Tlx1 loss itself did not significantly affect this perifollicular clustering of Venus^+^ cells (0.47 ± 0.02 in controls versus 0.49 ± 0.02 in Tlx1-deficient cells). Taken together, these findings indicate that Tlx1 expression in splenic mesenchymal stromal cells is required for recruiting HSPCs to the spleen, which then leads to the development of LPS-induced EMH.

## Discussion

Here, we have demonstrated that mesenchymal cells that express Tlx1 in the perifollicular region of the red pulp are an indispensable component of the HSPC niche in the postnatal spleen. We have also shown that the activation of Tlx1 expression in these cells ensures the recruitment and proliferation of HSPCs, leading to the development of splenic EMH. This conclusion is based on our findings that Tlx1 overexpression *in situ* in the spleen is able to induce EMH and the conditional loss of Tlx1 in these cells abolishes LPS-induced splenic EMH.

Mesenchymal stem/progeny cells (MSPCs) are recognized as a major component of the HSPC niche in the BM^31^, because they are an enriched source of CXL12 and SCF that are absolutely indispensable for HSPC maintenance and retention in the BM^32–35^. Among them, leptin receptor^+^ cells in the BM that express PDGFRα&β, CD51, CD105 are a major source of CXCL12 and SCF, as well as MSPC activity^36^, and are thought to overlap with CXCL12-abundant reticular (CAR) cells, which are primarily localized around sinusoids throughout the BM^37^. Although Tlx1-expressing cells lack leptin receptor expression, their cell surface phenotypes and functions otherwise resemble the MSPCs comprising the HSPC niche in the BM, as they express PDGFRα&β, CD51, CD105, and are a major source of CXCL12 and SCF in the spleen. In this regard, it has previous been demonstrated by using reporter mice that vascular sinusoidal cells and mesenchymal cells expressing Tcf21 located in the red pulp are the major source of SCF and that a subset of Tcf21-expressing cells express CXCL12^38^. Moreover, the conditional inactivation of SCF in each of these cell components and CXCL12 in Tcf21-expressing cells abolished EMH induced by myeloablation, blood loss and pregnancy. Based on our observation that Tlx1-expressing cells, compared to other splenic stromal cells, predominantly express both *SCF* and *CXCL12* mRNAs even in the steady state as well as after LPS treatment, and that the loss of Tlx1 in these cells reduced the mRNA levels of these HSPC niche factors to the levels seen in non Tlx1-expressing cells, Tlx1 expression appears to be not only a marker of MSPCs in the spleen, but also an upstream regulator of *SCF* and *CXCL12* gene expression. Thus, it seems likely that Tlx1-expressing cells overlap with Tcf21-expressing mesenchymal cells that express both CXCL12 and SCF.

With the exception of G-CSF, the activation of Tlx1 expression also appears to induce expression of various hematopoietic factors in MSPCs of the spleen, as Tlx1 overexpression in these cells up-regulates expression of not only HSPC niche factors but also M-CSF and BMP-4, which are known to participate in differentiation of monocytes and macrophages^28^ and of erythroid-lineage cells^26,27^, respectively. In this regard, the members of TALE family homeodomain transcription factors, including Pbx1, which function directly upstream of *Tlx1* gene expression in embryonic spleen mesenchymal anlage^19^, have recently been reported to participate in the normal and emergency hematopoiesis in the spleen^39^. Splenic mesenchymal cell-selective loss of *Pbx1* on a *Pbx2*- or *Pbx3*-deficient genetic background caused a profound decrease in numbers of HSPCs in the spleen and the mutant adult mice displayed impaired recovery of hematopoiesis after myelosuppressive irradiation. This hematopoietic abnormality caused by *Pbx* deficiency in the splenic stromal cells was associated with the reduced expression of genes encoding various hematopoietic factors, such as CXCL12, SCF, and GM-CSF. Although we have not examined in detail the effect of *Tlx1* loss on the expression of genes encoding hematopoietic factors that are affected by *Pbx* deficiency, there might be some overlap between Tlx1 and Pbx transcriptional targets. In line with a transcriptional network involved in the hematopoietic factor gene expression governed by Tlx1 and Pbx, Tlx3, a Tlx1 orthologue expressed in neurons, has been reported to cooperate with Pbx3 to recruit the epigenetic regulator CBP, leading to the expression of global glutamatergic neuronal genes^40^. Thus, it seems reasonable to assume that such a collaboration between Tlx1 with Pbx family members regulates expression of these hematopoietic factor genes in splenic mesenchymal cells.

The mobilization of HSPCs from the BM to the periphery is an essential process for EMH, which depends on the action of G-CSF^29,30^ accompanied by the effect of norepinephrine released form sympathetic nerves^41,42^. Our findings suggest that Tlx1-expressing cells are not the producers of G-CSF, instead they modulate indirectly the splenic HSPC niche components to produce G-CSF. We base this conclusion on our findings that serum G-CSF levels did not change at an early time point after Tlx1 overexpression or due to the loss of Tlx1, even in which LPS-induced elevation of G-CSF was augmented. In support of this notion, it has recently been reported that vascular endothelial cells are the main source of serum G-CSF in LPS-induced EMH, which was revealed by deleting the gene encoding Myd88, a downstream effector of toll-like receptor 4 (TLR4), specifically in Tie2-expressing cells^43^. Based on the previous finding that the vagus nerve-mediated elevation in serum norepinephrine levels is abolished by splenectomy^44^, the transient increase in LSK cells in the peripheral blood in the absence of serum G-CSF elevation upon Tlx1 overexpression might be due to the transient release of norepinephrine from noradrenergic nerve fibers that are abundantly distributed in the spleen^45^. Although direct evidence supporting this hypothesis is lacking, its potential involvement warrants further investigation.

Similar to the HSPC niche in the BM, which is a complex structure consisting not only of mesenchymal cells but also of nerve fibers^41,46^ and mature blood cells, such as megakaryocytes^47,48^ and macrophages^49,50^, and is dynamically modulated by the interaction of these niche components and inflammatory and neuronal signals from outside of the BM, the HSPC niche in the spleen must also have a similarly complex composition and regulation. With regard to the vascular and sympathetic nervous systems in the spleen, as mentioned above, the perifollicular region surrounding the white pulp represents the interface of blood flow into the sinusoids of the red pulp. The spleen is supplied with blood via the small branches of splenic central arterioles that terminate in the marginal sinusoid at the perifollicular area, and thereafter, the blood flow passes into venous sinusoid across the red pulp^51^. Thus, the perifollicular region in the spleen appears to be the most relevant site where circulating HSPCs initially make contact with mesenchymal cells as well as the vascular endothelial cells in the spleen. Besides vascular endothelial cells, the perifollicular area of the spleen is comprised of the marginal sinus lining cells, MAdCAM-1^+^ MRCs, that are surrounded by Tlx1-expressing mesenchymal cells in the red pulp and CD169^+^ macrophages inside the white pulp^6^, where noradrenergic nerve fibers innervate^45^. The chemical blockage of norepinephrine release from the nerve terminals has been reported to reduce CXCL13 expression in perifollicular CD31^−^ mesenchymal cells^52^, suggestive of sympathetic nervous system-mediated functional and/or structural modulation of the perifollicular area, as reported in the HSPC niche of the BM^53^. In addition, a study of CD169^+^ macrophage ablation also revealed that these cells participate in the splenic retention of HSPCs in LPS-induced EMH^54^. Thus, the accumulation of Tlx1-expressing cells at this area upon activation of Tlx1 expression might facilitate HSPC recruitment and lodging for EMH in the spleen via functionally interacting with other HSPC niche components, including marginal sinus endothelial cells, noradrenergic nerve fibers and CD169^+^ macrophages.

Further investigation of the role of Tlx1-expressing mesenchymal cells in creating the splenic HSPC niche for EMH and also of the upstream signals that modulate Tlx1 expression in splenic mesenchymal cells should provide important clues to understand homeostatic regulation of hematopoiesis balanced by events inside and outside of the BM under pathological conditions. Furthermore, the splenic HSPC niche, distinctly from the BM niche, has recently been demonstrated to be involved in the occurrence of chronic myeloid leukemia and myeloproliferative neoplasms^55,56^. Therefore, the splenic HSPC niche-selective EMH-inducing as well as -eliminating systems described in the present study could also be applied to dissect the relative contributions of BM- and splenic HSPC niches to myeloid malignancies.

## Methods

### Mice

Mice were housed in a specific pathogen-free facility, and all animal experiments were carried out in accordance with approved protocols of the Tokyo University of Science Animal Care and Use Committee. *Tlx1*^*CreER-Venus/+*^ mice have been described previously^25^. *R26*^*tdTomato* ^^57^ and *R26*^*DTA* ^^58^ mice (purchased from The Jackson Laboratories) were maintained on a C57BL/6-CD45.2 background. For the generation of the *R26*^*Tlx1*^ mouse strain, ES cells harboring a mutant *Rosa26* allele were generated by a recombinase-mediated cassette exchange method with the use of IDG3.2 Rosa26.10 ES cells and a cassette exchange vector (pEx-CAG-stop-bpA) inserted with HA epitope-tagged mouse *Tlx1* (*HA-Tlx1*) cDNA, according a protocol described previously^59^. In brief, the linearized pEx-CAGstop-HA-Tlx1-bpA vector was electroporated into IDG3.2 Rosa26.10 ES cells, with the pCAG-C3Int expression vector, and drug-resistant colonies were screened for homologous recombination. Targeted clones were injected into C57BL/6 blastocysts and the resultant chimeric mice were bred to produce progeny with germline transmission of the mutated allele. F1 progeny harboring a targeted *Rosa26* allele with *HA-Tlx1* cDNA (*R26*^*Tlx1*^) were then backcrossed onto the C57BL/6 background for 12 generations. The mouse strain carrying the floxed allele of the *Tlx1* gene (*Tlx1*^*fl*^) was generated as follows. In brief, the targeting vector containing a 3.7-kb genomic fragment immediately upstream of the *lox*P-flanked 0.95-kb fragment containing exon 1 of the *Tlx1* gene and 1.1-kb DNA fragment immediately downstream of the gene was electroporated into EGR-101 C57BL/6 strain-derived ES cells, and drug-resistant colonies were screened for homologous recombination. Targeted clones were injected into BALB/c blastocysts and the resultant chimeric mice were bred to produce progeny with germ line transmission of the mutated allele. F1 progeny harboring a targeted *Tlx1* allele were then crossed with ubiquitous *CAG*-promoter-driven FLPe mice on a C57BL/6 background (obtained from RIKEN Bioresource Center) to remove the FRT-flanked neomycin-resistant gene cassette. C57BL/6-CD45.1 mice were maintained in our laboratories^60^. Unless otherwise indicated, littermates were used as controls with indications of genotype and treatment in text or figure legends.

### *In vivo* treatments

Tamoxifen (0.1 g/kg body weight; Sigma-Aldrich, St Louis, MO) was delivered by intragastric gavage daily for 5 consecutive days to induce activation of CreER. LPS treatment was carried out by intraperitoneal injection of LPS (10 μg; E. coli O26, Wako Pure Chemical Industries) or vehicle (saline) for 3 consecutive days and analyses were performed on the fourth day. Peripheral blood count analysis was carried out as previously described^61^. For BrdU incorporation into HSPCs, mice were intraperitoneally injected with a single dose of 1 mg BrdU one hour before analyses. Cells stained with the indicated antibodies were fixed and stained with an anti-BrdU antibody using the FITC-BrdU Flow kit (BD Bioscience), according to the manufacturer’s protocol. In the case of BrdU incorporation analysis of Tlx1^+^ cells, mice were intraperitoneally injected with a single dose of 1 mg BrdU and maintained on 1 mg/ml of BrdU in the drinking water for 10 days. When indicated, sorted 10000 LSK cells from BM of C57BL/6-CD45.1 mice were transplanted via intravenous injection into lethally irradiated (8 Gray) mice. Four hours after transplantation, the recipient mice were analyzed for the presence of CD45.1 positive cells by flow cytometry. The level of serum G-CSF was measured using an ELISA kit (R&D system) according to the protocol provided by the kit.

### Cell preparations

Cell preparations for the analysis of hematopoietic cell populations were carried out as previously described^60^. For stromal cell preparation, a whole spleen was transferred into 100 μl of RPMI-1640 medium (Wako Pure Chemical Industries), and was cut and minced using scissors. After adding 900 μl of RPMI and vortexing, the spleen fragments were allowed to sediment for 1 min and the supernatant was transferred to another tube on ice. This process was repeated, collecting the supernatant each time. After collecting the hematopoietic cells, spleen capsules were digested in RPMI-1640 medium containing 4.0 mg/ml collagenase (Wako Pure Chemical Industries) with 0.1 mg/ml DNaseI (Sigma-Aldrich), and incubated for 20 min at 37 °C, with tapping every 10 min. The supernatant was transferred to another tube containing 3 ml of MACS buffer on ice. This process was repeated twice, collecting the supernatant each time. The residual sediments were homogenized by using a 1 ml syringe fitted with a 27-gauge needle (Terumo). The preparation of lymph node, thymus and liver stromal cells was also performed by the protocol as described above. For the bone marrow stromal cells, femurs and tibias were dissected and crushed with a pestle. The crushed bones were gently washed once in PBS, and the cell suspension filtered through a cell strainer (Falcon 2350) was discarded. The bone fragments were further cut using scissors, then transferred into new tube. The bone fragments were gently washed in PBS, then incubated for 1 hour at 37 °C in 20 ml of RPMI-1640 medium containing 0.25% collagenase. The suspension was filtered with a cell strainer to remove debris and bone fragments, and was collected by centrifugation, as previously described^62^.

### Flow cytometry and cell sorting

Single cell suspensions from the indicated organs were stained with indicated antibodies and data were collected on FACSCalibur or FACSCanto^TM^II flow cytometers (BD Bioscience) and analyzed using FlowJo software (TreeStar, Ashland, OR). A FACSArea^TM^II (BD Bioscience) was used for cell sorting. Lineage markers (Lin) for LSK and LK cell populations were composed of CD3, CD11b, B220, Gr-1, NK1.1 and Ter119. Lin markers for stromal cell analysis were composed of CD45.2, Ter119 and CD31. Antibodies used for flow cytometry and sorting are listed in Table S1.

### Immunohistochemistry

Freshly dissected spleen was fixed in 4% paraformaldehyde overnight followed by incubation gradually in 10%, 20% and then 30% sucrose in PBS. Frozen spleens embedded in OTC compound (Sakura Finetek Japan) were serially sectioned (8 μm) with a cryostat (Leica Camera). Sections were blocked, stained with antibodies and mounted with anti-fade prolong gold (Thermo Fisher Scientific). Images were acquired with either a BIOREVO BZ9000 (KEYENCE) or a confocal laser-scanning microscope (FV1000-D, Olympus Corporation). Lin markers for HSPC analysis were composed of CD3, CD11b, B220, Gr-1, NK1.1 CD71 and Ter119. Staining antibodies are listed in Table S2.

### Image processing and analyses

Images were processed with ImageJ (http://imagej.nih.gov/ij/) and Imaris 7.2.1 (Bitplane) software. Brightness adjustments and thresholds were applied equally to images captured from experimental and control samples. For clustering analyses, digital images were analyzed with ImageJ-plugin software Particle Picker (http://bigwww.epfl.ch/thevenaz/pointpicker/) customized to find and count coordinates of Venus-positive cells. Data were exported in Microsoft Office 2016 Excel software, and were calculated using custom-made clustering algorithm based on clustertend (http://cran.r-project.org/package=clustertend). Randomly chosen individual subsets of 40 localizations each were analyzed 1000 times within that window (150 μm × 150 μm) from at least 3-independent samples to evaluate the Hopkins Index. Quantitative assessment of the cell distributions was carried out using the Imaris XT module which integrates MATLAB applications (R2016a(9.0.0.341360); MathWorks). The splenic specimens (a typical volume of 600 × 600 pixels) were examined regarding cell numbers, positions and relationships to each other by distance transformation (http://open.bitplane.com/tabid/235/Default.aspx?id=17). Statistics were exported in GraphPad Prism7. Data were compared with the randomly distributed points of equal density generated using custom-made code in MATLAB.

### Quantitative PCR

Total RNA was extracted with a ReliaPrep RNA cell Miniprep System (Promega Corporation), according to the manufacturer’s instructions. The cDNAs were synthesized using Superscript VILO cDNA synthesis system (Thermo Fisher Scientific). Real-time PCR was performed using SYBER Premix Ex Taq (Takara) and CFX384 Real-time System (Bio-Rad). The primer sequences are listed in Table S3.

### Statistical analysis

The numbers of mice used for each experiment are shown in the Figure legends. Differences between groups were evaluated using Prism 7 software (GraphPad Software, La Jolla, CA). Values are expressed as mean ± standard deviation unless otherwise indicated, and statistical significances were compared with 2-tailed Student’s t test for 2 groups, one-way ANOVA with Dunnet’s test for multiple comparison. A *P* value of less than 0.05 was considered significant.

### Data availability

The authors declare that all other data supporting the findings of this study are available within the article and its supplementary information files.

## Electronic supplementary material

Supplementary information
