# Supplementary material for: Niche-induced extramedullary hematopoiesis in the spleen is regulated by the transcription factor Tlx1
Source: Sci Rep. 2018 May 29;8:8308. doi: 10.1038/s41598-018-26693-x (PMC5974313; doi:10.1038/s41598-018-26693-x)
Supplement: Supplementary file 1 — Supplementary information [file 41598_2018_26693_MOESM1_ESM.pdf]

Niche-induced extramedullary hematopoiesis in the spleen is regulated by the transcription factor Tlx1

Akihisa Oda[1], Toshiki Tezuka[1], Yuta Ueno[1,2], Shoko Hosoda[1,2], Yusuke Amemiya[1,2], Chihiro Notsu[1], Toru Kasahara[1], Chiharu Nishiyama[2] and Ryo Goitsuka[1,3,4,*]

[1]Division of Development and Aging, Research Institute for Biomedical Sciences, Tokyo University of Science, Chiba, Japan
[2]Laboratory of Molecular Biology and Immunology, Department of Biological Science and Technology, Tokyo University of Science, Tokyo, Japan
[3]Center for Animal Disease Models, Tokyo University of Science, Chiba, Japan
[4]Imaging Frontier Center, Tokyo University of Science, Chiba, Japan

[*]Corresponding author:
Ryo Goitsuka, Ph.D., D.V.M., Division of Development and Aging, Research Institute for Biomedical Sciences, Tokyo University of Science, 2669 Yamazaki, Noda, Chiba 278-0022, Japan; e-mail: ryogoi@rs.noda.tus.ac.jp

Key words: Niche, hematopoietic stem/progenitor cells, spleen, mesenchymal cells

Supplementary Figure Legends

Fig. S1 Tlx1 is not expressed in the hematopoietic and endothelial compartment of the spleen. Representative flow cytometry gating schemes for the stromal cell compartment and the hematopoietic and endothelial compartment of the spleen from $Tlx1^{CreER\text{-}Venus}$; $R26^{tdTomato}$ mice treated with or without tamoxifen (Tx).

Fig. S2 Effect of Tlx1 overexpression on hematopoiesis in the spleen and BM. $Tlx1^{CreER\text{-}Venus}$ littermate controls and $Tlx1^{CreER\text{-}Venus}$;$R26^{Tlx1}$ mice (4-week-old) were treated with tamoxifen and hematopoietic cell populations in the spleen and BM were analyzed 4 weeks after the final treatment. Data were pooled from 2-4 independent experiments.
(**a**) Gross appearance (left), the weight (middle) and cell numbers (right) of the spleen from tamoxifen-treated $Tlx1^{CreER\text{-}Venus}$;$R26^{Tlx1}$ mice (Tg), compared to the spleen from tamoxifen-treated $Tlx1^{CreER\text{-}Venus}$ littermates (Ctr). (mean±SD, n=5). (**b**) Total numbers of the indicated mature hematopoietic cell populations from the spleen of tamoxifen-treated $Tlx1^{CreER\text{-}Venus}$ controls (Ctr) and tamoxifen-treated $Tlx1^{CreER\text{-}Venus}$;$R26^{Tlx1}$ mice (Tg). (mean±SD; n = 6). (**c**) Total numbers of the indicated hematopoietic stem/progenitor cell populations from the spleen of tamoxifen-treated $Tlx1^{CreER\text{-}Venus}$ controls (Ctr) and tamoxifen-treated $Tlx1^{CreER\text{-}Venus}$;$R26^{Tlx1}$ mice (Tg). (mean±SD; n = 3-6). (**d**) Total numbers of the indicated hematopoietic stem/progenitor cell populations in the BM of tamoxifen-treated $Tlx1^{CreER\text{-}Venus}$ controls (Ctr) and tamoxifen-treated $Tlx1^{CreER\text{-}Venus}$;$R26^{Tlx1}$ mice (Tg). (mean±SD; n = 6). (**e**) Total numbers of the indicated mature hematopoietic cell populations in the BM of tamoxifen-treated $Tlx1^{CreER\text{-}Venus}$ controls (Ctr) and tamoxifen-treated $Tlx1^{CreER\text{-}Venus}$;$R26^{Tlx1}$ mice (Tg). (mean±SD; n = 6).

Fig. S3 Generation of the conditional *Tlx1* allele. (a) Diagram depicting the *Tlx1* locus and the targeting strategy used to generate mutant *Tlx1* alleles (floxed and deleted alleles). *LoxP* sites (arrowheads) were inserted into intronic sites flanking exon 1 of the

Tlx1 gene. The FRT-flanked neomycin gene (PGK-neo) selection cassette was removed by crossing to CAG-FLPe mice. PCR primers for verifying the Cre-mediated deletion of the *lox*P-flanked fragment are indicated by arrows. DT-A, a diphtheria toxin negative selection cassette; DT-A, a diphtheria toxin negative selection cassette; E, *Eco*RI; S, S*ca*I. (**b)** The correct integration of the targeting vector was verified by Southern blot analysis of *Sca*I- or *Eco*RI-digested genomic DNA from ES cells using the indicated probe in (**a**). (**c)** Confirmation of *Tlx1* deletion. Genomic DNAs from spleen stromal cells of wild-type littermates (*Tlx1*$^{+/+}$) and *Tlx1*$^{CreER\text{-}Venus/fl}$ mice as well as Venus^{+} and Venus^{-} spleen stromal cells of tamoxifen-treated *Tlx1*$^{CreER\text{-}Venus/fl}$ mice were subjected to PCR analysis using the indicated primer pairs in (**a**). The sequence of primers is as follows; 5'-TCTGTCTGTTCGTCTTTGGG-3' for primer 1 and 5'-ACAACTCAGAGCACGTGTG-3' for primer 2. M, DNA size marker.

Fig. S4 Full blot image of Fig. S3b.

Fig. S5 Full gel image of Fig. S3c.

Figure S1

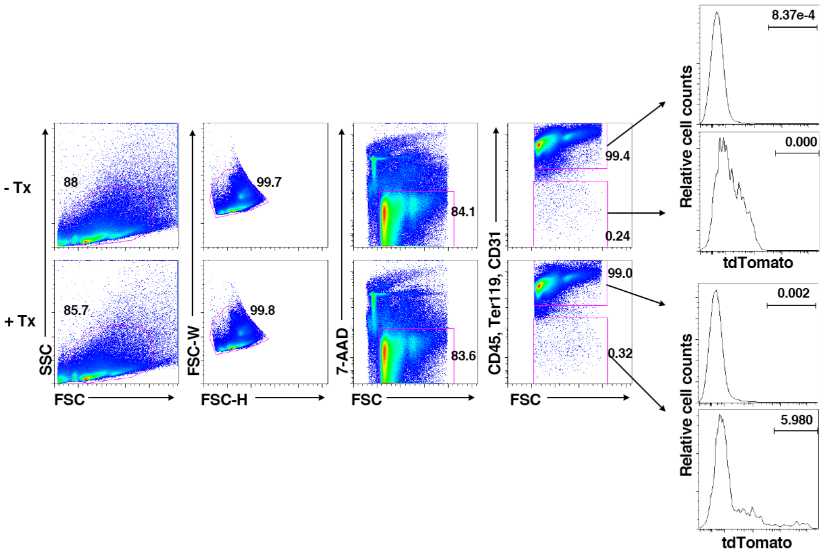

- Tx
+ Tx
88
85.7
SSC
FSC
99.7
99.8
FSC-W
FSC-H
84.1
83.6
7-AAD
FSC
99.4
0.24
99.0
0.32
CD45, Ter119, CD31
FSC
8.37e-4
0.000
0.002
5.980
Relative cell counts
Relative cell counts
tdTomato
tdTomato

Figure S2

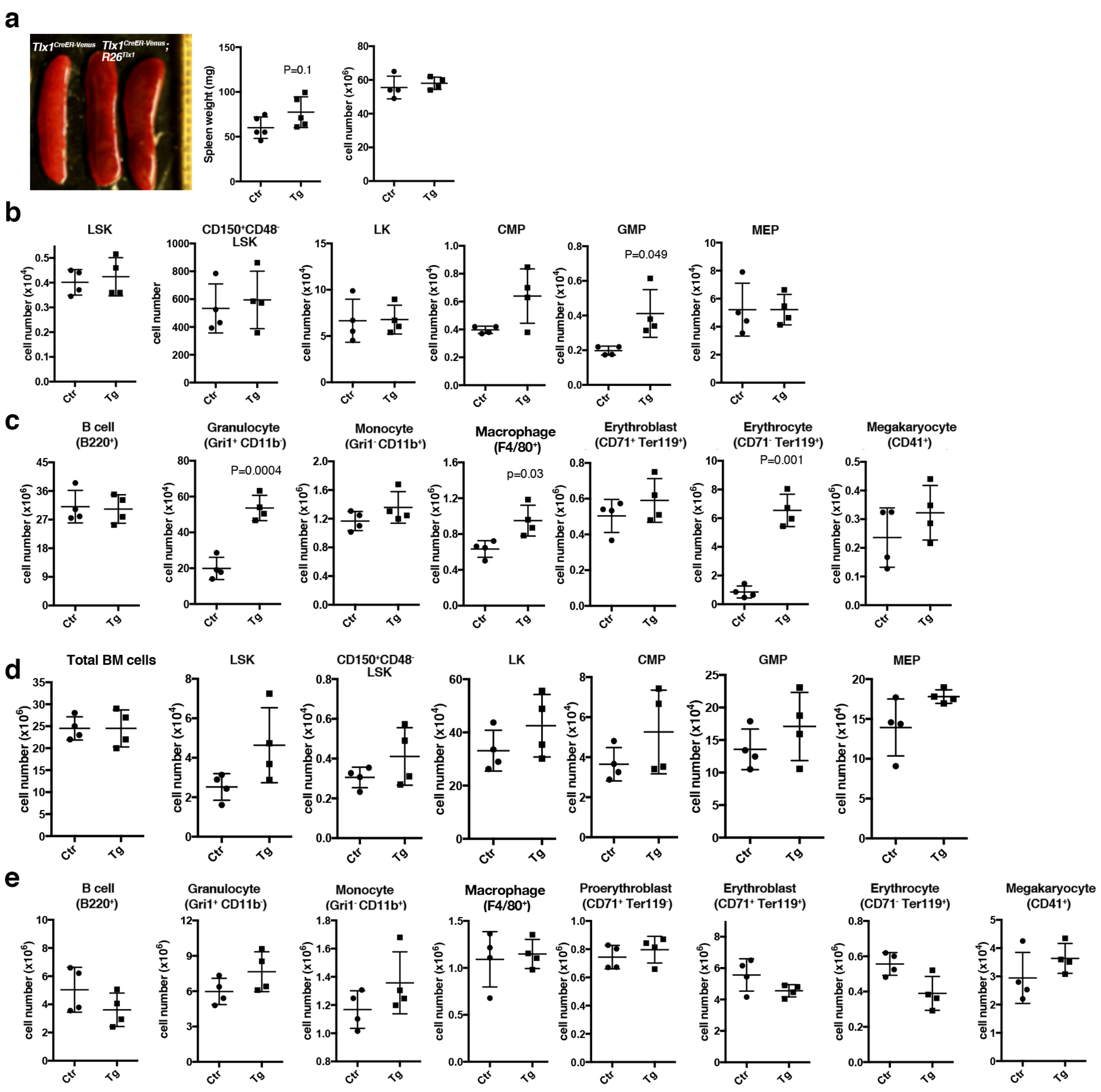

a
Tlx1CreER-Venus
Tlx1CreER-Venus; R26Tlx1
P=0.1
Spleen weight (mg)
cell number (x10⁶)
b
LSK
CD150+CD48- LSK
LK
CMP
GMP
MEP
P=0.049
c
B cell (B220+)
Granulocyte (Gri1+ CD11b-)
Monocyte (Gri1- CD11b+)
Macrophage (F4/80+)
Erythroblast (CD71+ Ter119+)
Erythrocyte (CD71- Ter119+)
Megakaryocyte (CD41+)
P=0.0004
p=0.03
P=0.001
d
Total BM cells
LSK
CD150+CD48- LSK
LK
CMP
GMP
MEP
e
B cell (B220+)
Granulocyte (Gri1+ CD11b-)
Monocyte (Gri1- CD11b+)
Macrophage (F4/80+)
Proerythroblast (CD71+ Ter119-)
Erythroblast (CD71+ Ter119+)
Erythrocyte (CD71- Ter119+)
Megakaryocyte (CD41+)
Ctr
Tg
cell number

Figure S3

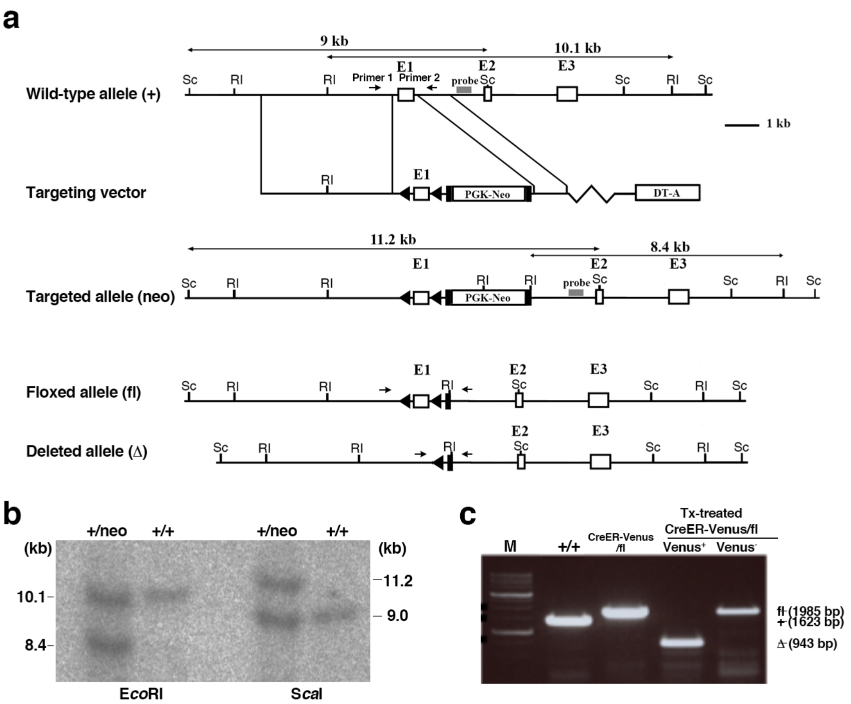
a
9 kb
10.1 kb
Wild-type allele (+)
Sc
RI
RI
Primer 1
E1
Primer 2
probe
E2
Sc
E3
Sc
RI
Sc
1 kb
Targeting vector
RI
E1
PGK-Neo
DT-A
11.2 kb
8.4 kb
Targeted allele (neo)
E1
E2
E3
probe
Floxed allele (fl)
Deleted allele (Δ)
b
+/neo
+/+
(kb)
10.1
8.4
11.2
9.0
EcoRI
ScaI
c
M
CreER-Venus /fl
Tx-treated CreER-Venus/fl
Venus+
Venus-
fl (1985 bp)
+ (1623 bp)
Δ (943 bp)

Figure S4

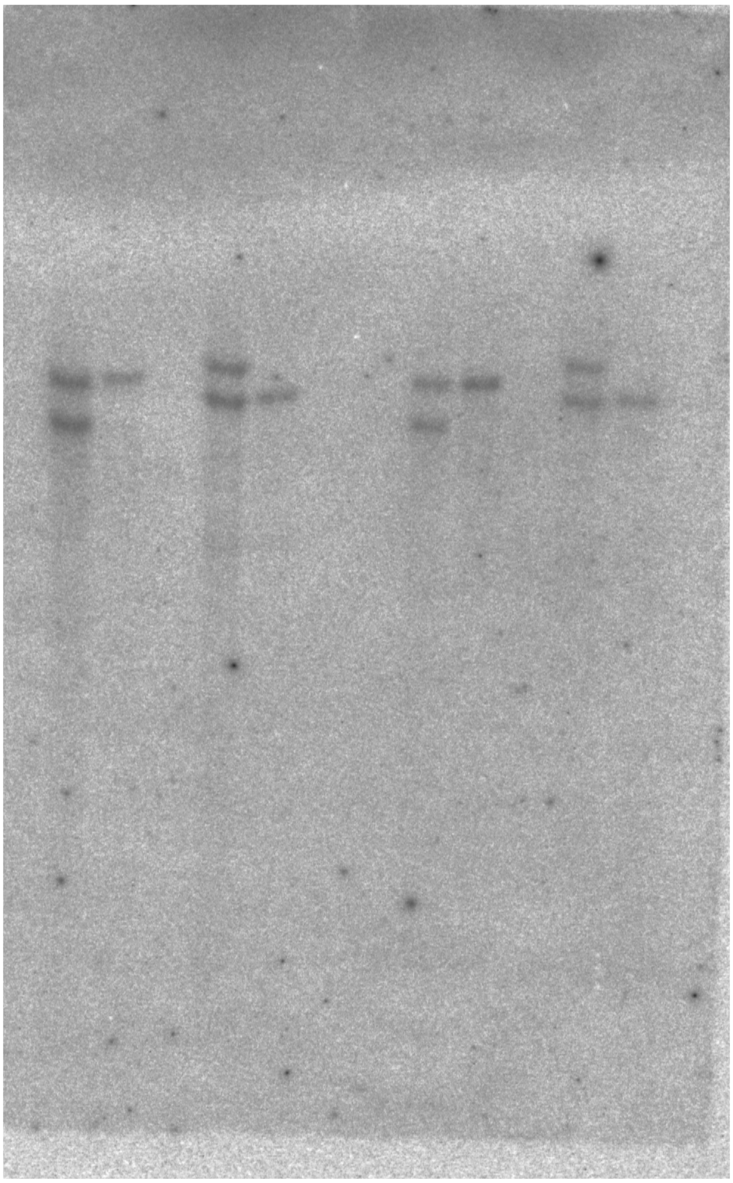

Figure S5

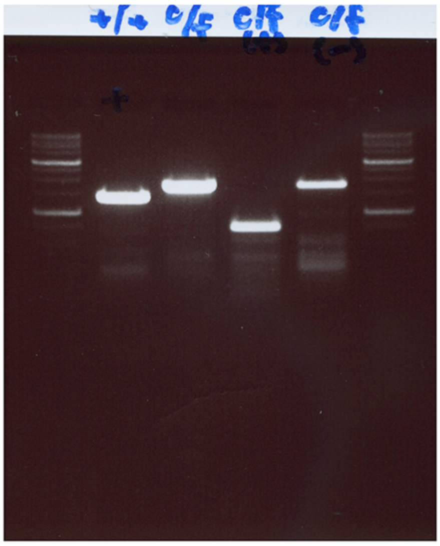

Table S1. Antibodies used for flow cytometry

Antibody	Clone	Conjugated	Company
B220	RA3-6B2	PE	BioLegend
B220	RA3-6B2	PerCP/Cy5.5	BioLegend
CD3ε	145-2C11	FITC	BioLegend
CD3ε	145-2C11	PerCP/Cy5.5	BioLegend
CD11b	M1/70	PE	eBioscience
CD11b	M1/70	PerCP/Cy5.5	BioLegend
CD16/32	93	PE	BioLegend
CD21/35	·7E9	FITC	BioLegend
CD21/35	·7E9	APC	BioLegend
CD23	B3B4	PE	BioLegend
CD29	eBioHMb1-1	PE-Cy7	eBioscience
CD31	390	Alexa647	BioLegend
CD31	390	PE	BioLegend
CD34	RAM34	eFluor 660	eBioscience
CD41	MWReg30	PE	BD Biosciences
CD44	IM7	PE-Cy7	eBioscience
CD45.2	104	PerCP/Cy5.5	BioLegend
CD45.2	104	Brilliant Violet 421	BioLegend
CD48	HM48-1	Pacific Blue	BioLegend
CD51	RMV7	Biotin	BioLegend
CD71	C2	BV421	BD Biosciences
CD71	C2	PerCP/Cy5.5	BD Biosciences
CD105	MJ7/18	Biotin	BioLegend
CD117 (c-kit)	ACK2	APC	BioLegend
CD117 (c-kit)	2B8	APC/Cy7	BioLegend
CD140a	APA5	APC	eBioscience
CD140b	APB5	APC	BioLegend
CD150	TC15-12F12.2	PE	BioLegend
F4/80	BM8	FITC	eBioscience
IgM	RMM-1	APC	BioLegend
LeptinR	polyclonal	Biotin	R&D SYSTEM
LtbR	3C8	Biotin	eBioscience
Ly-6G/Ly-6C	RB6-8C5	Pacific Blue	BioLegend
Ly-6G/Ly-6C	RB6-8C5	PerCP/Cy5.5	BioLegend
MAdCAM1	MECA367	PE	Santa Cruz Biotechnology
MAdCAM1	MECA367	Biotin	BioLegend
NK1.1	PK136	PerCP/Cy5.5	eBioscience
Podoplanin	8.1.1	Biotin	BioLegend
Sca-1	D7	FITC	eBioscience
TCRβ	H57-597	FITC	BioLegend
Ter119	TER-119	APC	BioLegend
Ter119	TER-119	PerCP/Cy5.5	BioLegend
Ter119	TER-119	Pacific Blue	BioLegend
VCAM1	429	Alexa Fluor 647	BioLegend
VEGFR2	Avas12	APC	BioLegend
Arm Ham IgG	eBio299Arm	PE-Cy7	eBioscience
Arm Ham IgG	HTK888	Biotin	BioLegend
Syrian Ham IgG	SHG-1	Biotin	BioLegend
Goat IgG	ab37376	Biotin	abcam
Rat IgG2a	17-4321	APC	eBioscience
Rat IgG2a	RTK2758	Biotin	BioLegend
Rat IgG2a	sc-2872	PE	Santa Cruz Biotechnology
Rat IgG1	RTK2071	Biotin	BioLegend
Streptavidin		APC	eBioscience
Streptavidin		eFluor 450	eBioscience

Table S2. Antibodies used for immunohistochemical analysis

Antibody	Clone	Conjugated	Company
GFP	Rabbit polyclonal	Purified	MBL
Rabbit IgG	Donkey polyclonal	Alexa Fluor 488	Thermo Fisher
Rabbit IgG	Goat polyclonal	Alexa Fluor 647	Cell signaling
CD3ε	145-2C11	PE	BioLegend
CD3ε	145-2C11	APC	BioLegend
CD3ε	145-2C11	Biotin	BioLegend
B220	RA3-6B2	PE	BioLegend
B220	RA3-6B2	APC	BioLegend
B220	RA3-6B2	Biotin	BioLegend
Podoplanin	8.1.1	Biotin	BioLegend
MAdCAM1	MECA367	Biotin	BioLegend
CD21/35	･7E9	Biotin	BioLegend
ERTR7	ER-TR7	Purified	abcam
CD31	390	Alexa647	BioLegend
aSMA	1A4	Cy3	SIGMA
CD71	C2	Biotin	BD Bioscience
F4/80	BM8	Biotin	BioLegend
FDCM1	FDCM1	Purified	BD Bioscience
CD41	MWReg30	Alexa Fluor 488	BioLegend
CD150	TC15-12F12.2	PE	BioLegend
Ter119	Ter119	Biotin	BioLegend
CD11b	M1/70	Biotin	BioLegend
NK1.1	PK136	Biotin	BioLegend
Gr1	RB6-8C5	Biotin	eBioscience
Streptavidin		Alexa Fluor 488	Thermo Fisher
Streptavidin		Alexa Fluor 548	Thermo Fisher
Streptavidin		Alexa Fluor 633	Thermo Fisher

Table S3. Primers used for real-time PCR

β-actin-s	5'-GGCTGTATTCCCCTCCATCG-3'
β-actin-as	5'-CCAGTTGGTAACAATGCCATGT-3'
SCF-s	5'-TCTGCGGGAATCCTGTGACT-3'
SCF-as	5'-CGGCGACATAGTTGAGGGTTAT-3'
M-CSF-s	5'-GAGGTGTCAGAACACTGTAG-3'
M-CSF-as	5'-CAATCTGGCATGAAGTCTCC-3'
BMP4-s	5'-TTCCTGGTAACCGAATGCTGA-3'
BMP4-as	5'-CCTGAATCTCGGCGACTTTTT-3'
CXCL12-s	5'-GCTCTGCATCAGTGACGGTA-3'
CXCL12-as	5'-TAATTTCGGGTCAATGCACA-3'
mTlx1 Ex-s	5'-CGCAGATACACAAAGGACAG-3'
mTlx1 Ex-as	5'-TTTCCAGCTCACAGATCTGC-3'
